# Mechanistic Understanding of the Interactions of Cationic Conjugated Oligo- and Polyelectrolytes with Wild-type and Ampicillin-resistant *Escherichia coli*

**DOI:** 10.1038/s41598-019-56946-2

**Published:** 2019-12-31

**Authors:** Ehsan Zamani, Shyambo Chatterjee, Taity Changa, Cheryl Immethun, Anandakumar Sarella, Rajib Saha, Shudipto Konika Dishari

**Affiliations:** 1 Department of Chemical and Biomolecular Engineering, University of Nebraska-Lincoln, Lincoln, Nebraska 68588 United States; 2 Nebraska Center for Materials and Nanoscience, Voelte-Keegan Nanoscience Research Center, University of Nebraska-Lincoln, Lincoln, NE 68588-0298 United States

**Keywords:** Nanoscale materials, Soft materials, Conjugated polymers

## Abstract

An in-depth understanding of cell-drug binding modes and action mechanisms can potentially guide the future design of novel drugs and antimicrobial materials and help to combat antibiotic resistance. Light-harvesting π-conjugated molecules have been demonstrated for their antimicrobial effects, but their impact on bacterial outer cell envelope needs to be studied in detail. Here, we synthesized poly(phenylene) based model cationic conjugated oligo- (2QA-CCOE, 4QA-CCOE) and polyelectrolytes (CCPE), and systematically explored their interactions with the outer cell membrane of wild-type and ampicillin (amp)-resistant Gram-negative bacteria, *Escherichia coli* (*E. coli*). Incubation of the *E. coli* cells in CCOE/CCPE solution inhibited the subsequent bacterial growth in LB media. About 99% growth inhibition was achieved if amp-resistant *E. coli* was treated for ~3–5 min, 1 h and 6 h with 100 μM of CCPE, 4QA-CCOE, and 2QA-CCOE solutions, respectively. Interestingly, these CCPE and CCOEs inhibited the growth of both wild-type and amp-resistant *E. coli* to a similar extent. A large surface charge reversal of bacteria upon treatment with CCPE suggested the formation of a coating of CCPE on the outer surface of bacteria; while a low reversal of bacterial surface charge suggested intercalation of CCOEs within the lipid bilayer of bacteria.

## Introduction

Antibiotic resistance is an emerging global concern that requires immediate attention. In addition to regulating the use of the antibiotics, we need to focus on the development of alternate antibiotics based on a solid understanding of the drug-cell interactions. The cell walls of many Gram-negative bacteria play a critical role in modulating antibiotic resistance. The cell wall of *E. coli*, a Gram-negative bacterium, consists of an outer^1^ and inner membrane separated by a periplasmic space. The major constituents of the lipid bilayer^2^ on the outer membrane of Gram-negative bacteria are lipopolysaccharides (LPSs) having zwitterionic core oligosaccharides^3,4^; saturated fatty acid chains with zwitterionic phospholipid head groups^5^, and, lipid A functionalized with anionic phosphate groups^6–8^. The outer membrane also consists of porins^9^ which are proteins (charged) that allow the penetration of drugs, nutrients and small molecules inside bacterial cells. The periplasmic space under the lipid bilayer of outer membrane contains cross-linked peptidoglycans^5^. β-lactam antibiotics (such as ampicillin), when in action on wild-type *E. coli*, are able to penetrate through porins present on the outer cell envelope and reach to periplasmic space where the drugs inhibit the formation of peptidoglycan cross-linkages and block the cell division^10,11^. The most common ways Gram-negative bacteria show antibiotic resistance are: (i) by cleaving the antibiotic drugs (such as β-lactam) in the periplasmic space through secretion of enzymes (such as β-lactamase)^12^; (ii) by decreasing the number and size of the porins facilitating drug transport^13,14^; and, (iii) by altering the preferential permeation of certain species and transverse electrostatic field^15^ within the constriction zones of porins where the antibiotic docks before translocation inside the cells. These facts suggest that the charged nature of the outer cell envelope can play critical roles in the electrostatic binding interactions, transport of charged molecules, and ultimate killing/inhibitory actions of the drugs on both wild-type and antibiotic-resistant bacteria.

While β-lactams are widely used to treat infections associated with Gram-negative bacteria, the emergence of resistance to this class of antibiotics has led researchers to find alternative drugs. The two major classes of alternate candidates are cationic, gene-encoded antimicrobial peptides (AMPs)^16–20^, and, π-conjugated oligo/polyelectrolytes^18,21–27^. Initial studies suggested that bacteria still find it difficult to show resistance against the second class of antimicrobial molecules^24^. Conjugated molecules have unique light-harvesting, π-conjugated and hydrophobic backbones with charged functional groups as pendants. Cationic, water-soluble oligo- (CCOEs) and polyelectrolytes (CCPEs) can thus bind to the bacterial cell wall through electrostatic and hydrophobic interactions. In the efforts of designing small (CCOEs) and large (CCPEs)conjugated molecules, phenylene vinylene^26,28^, phenylene ethynylene ^18,29^, fluorene^22,30^, or thiophene^22,31^ are commonly used as backbone repeat units (RUs), while quaternary amine groups are used to impart positively charged functionalities to the conjugated molecules. The antimicrobial efficiency was influenced by the location and density of charged groups, backbone structure, chain length, and, the water solubility of CCOEs and CCPEs ^6,18,21,23,28,29^. Shorter side-chain CCOEs often appeared to be more toxic (lower minimum inhibitory concentration) to bacteria due to their intercalation within the lipid bilayer of the bacterial cell wall^6,21,28,29,32,33^. On the other hand, some other dominant antimicrobial actions (by CCOEs/CCPEs) took place *via* coating^18,23,30^, disruption of the lipid bilayer^18,32^, disintegration of the bacterial cytoplasmic membrane (bacteriolysis)^23^, and/or formation of reactive oxygen species (light-assisted)^27,34,35^. Combination therapies were also proposed where AMPs and CCPEs acted synergistically^22,30^.

**Figure 1:**
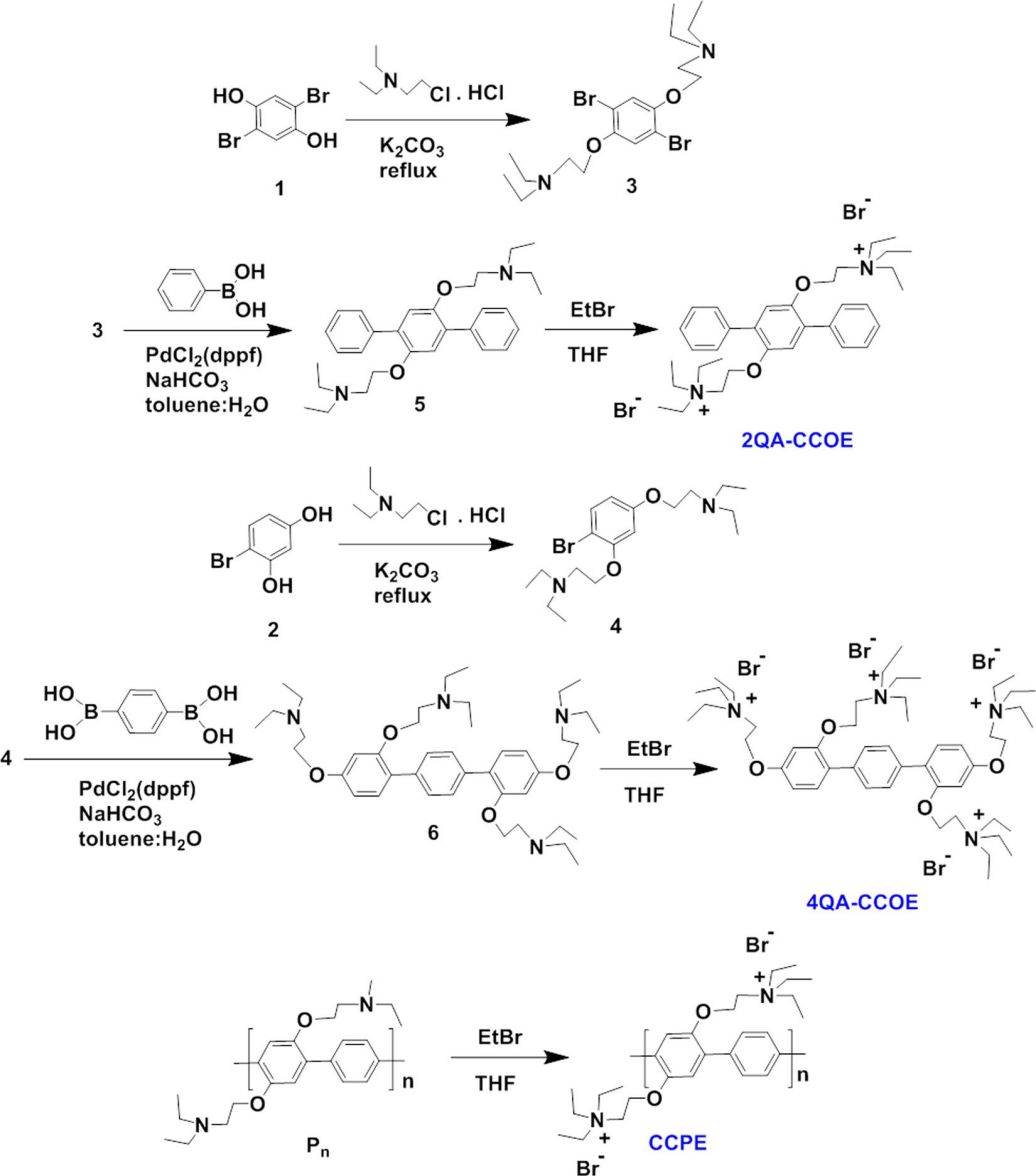
Synthetic routes to cationic conjugated oligoelectrolytes (2QA-CCOE, 4QA-CCOE) and polyelectrolyte (CCPE).

While phenylene vinylene^26,28^, and phenylene ethynylene^18,29^ based conjugated molecules have been studied to a great extent, the physical alterations of the bacterial outer membrane upon treatment with phenylene based cationic conjugated oligo- and polyelectrolytes are still underexplored. With a simplified backbone structure, phenylene based conjugated molecules can be ideal to explore the fundamental interactions with bacterial cells. In this work, we thus synthesized model cationic π-conjugated oligoelectrolytes (2QA-CCOE and 4QA-CCOE) and polyelectrolyte (CCPE) with phenylene based hydrophobic backbone and propoxy pendants terminated with cationic quaternary amine groups (Fig. 1). The ratios of phenyl groups to quaternary amine groups in CCPE, 2QA-CCOE, and 4QA-CCOE were 1: 1, 3: 2, and, 3: 4, respectively. Prior reports showed evidence of changes in cell elasticity, hydrophilic/hydrophobic interactions, nanoscale morphology, and membrane permeability when β-lactam resistant *E. coli* were treated with ampicillin^36^. Even different strains of amp-resistant *E. coli* can exhibit different phenotypical traits^36^. Since the cell-drug interaction is highly dependent on the type of strains as well as the drug molecules used, it is critical to explore the interactions of conjugated small and large molecules with both wild-type and amp-resistant bacterial strains side-by-side. In this work, we explored the antimicrobial activity of conjugated 2QA-CCOE, 4QA-CCOE, and CCPE molecules against wild-type (DH10B) and amp-resistant (SSBIO002) (explained in the supporting information) Gram-negative *E. coli* for a complex matrix of treatment conditions, including chemical structure and concentration of CCOE/CCPE in treatment systems as well as time and modes of treatments of bacterial cells. The killing efficiency (UV/Vis absorbance and flow cytometry); % growth inhibition (UV/Vis absorbance and colony-forming unit (CFU) assays); surface charge reversal (zeta potential analyzer); and, morphological changes (scanning electron microscopy (SEM), and fluorescence microscopy) of treated bacteria were explored. Such rigorous effort can greatly clarify our understanding of cell-conjugated molecule binding and growth inhibition mechanism from the viewpoint of fundamental changes on the bacterial membrane. This will also provide an informative framework for the rational design of antibiotics, and fight against antibiotic resistance. Besides aiding in drug design, this effort to unravel bacteria-organic molecule interactions can assist in the development of coating for medical devices, wound healing materials, packaging materials for food safety, prevention of marine biofouling, wastewater treatment, microbial sensing, microbial fuel cells and more.

## Results and Discussion

### Synthesis and optical properties of CCOEs and CCPE

For this specific study, we synthesized two phenylene based model oligoelectrolytes each with 3 phenyl groups along the backbone, and two (2QA-CCOE) or four (4QA-CCOE) propoxy pendants terminated with cationic quaternary amine groups (Fig. 1). The CCOEs were synthesized via Suzuki coupling and subsequent quaternization reaction (using ethyl bromide). We also synthesized cationic conjugated polyelectrolyte (CCPE) with phenylene based backbone following the procedure described in literature^37^ (Fig. 1). The step-by-step details of chemical synthesis and characterization to confirm the chemical structures of CCOEs and CCPE are discussed in supplementary information.

**Figure 2:**
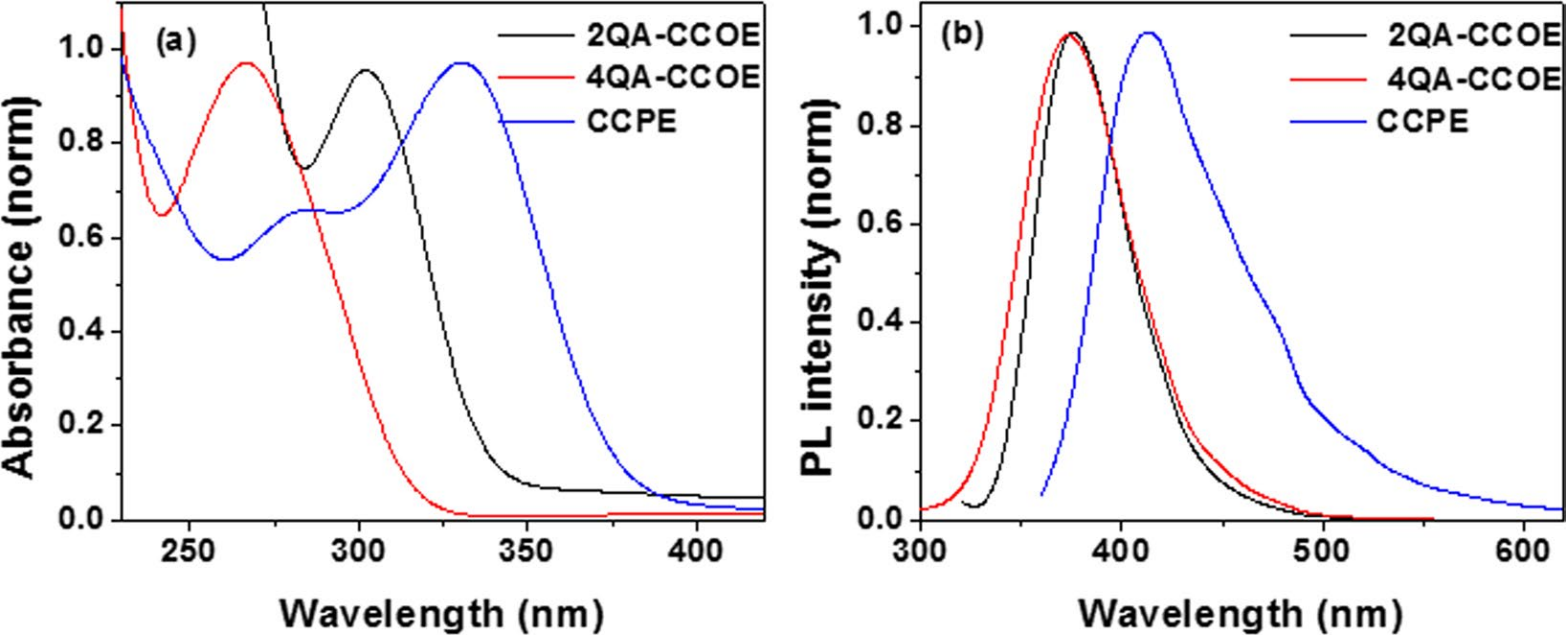
UV/Vis absorbance (**a**) and fluorescence (**b**) spectra of 2QA-CCOE, 4QA-CCOE, and CCPE in water.

The absorbance maxima for 2QA-CCOE and 4QA-CCOE in water were at 301 nm and 266 nm, respectively (Fig. 2a). Since the larger number of cationic quaternary amine groups in 4QA-CCOE structure rendered higher water solubility, 4QA-CCOE showed an absorbance maxima blue-shifted from 2QA-CCOE. CCPE, on the other hand, showed structured absorption spectra with two peaks at 283 nm and 330 nm. A higher conjugation length of CCPE led to the emission maxima red-shifted from the CCOEs (Fig. 2b, λ_em, max_ = 375, 374, 413 nm, for 2QA-CCOE, 4QA-CCOE and CCPE, respectively).

### Treatment of *E. coli* with CCPE/CCOE

The wild-type *E. coli* strain (DH10B) was transformed by appropriate plasmids to yield ampicillin-resistant (amp-resistant) *E. coli* strains (SSBIO002, see the experimental section, Supplementary Table S1 and other supporting information for details)^38,39^. Here, we treated *E. coli* suspension (wild-type and amp-resistant) with conjugated molecules in multiple ways. We first followed *two-step processes* where the cells were incubated with conjugated molecules first (either in phosphate-buffered saline (PBS) or Luria Bertani (LB) medium) and then transferred to LB media to allow the bacteria grow further (treat (PBS or LB), then grow (LB)). In addition, we followed a *one-step process* where the *E. coli* cells were treated and grown at the same time in LB media (treat and grow (LB)).

**Figure 3:**
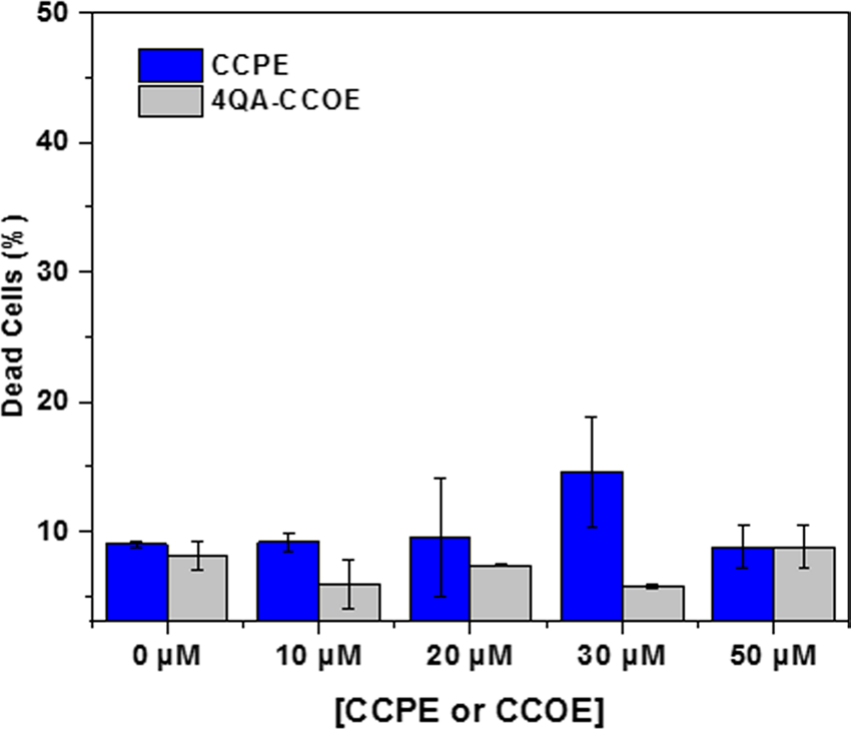
Live/dead bacterial assay of wild-type *E. coli* treated with 4QA-CCOE and CCPE in PBS buffer. Each data point represents the mean and standard deviation of 2 replicates.

**Figure 4:**
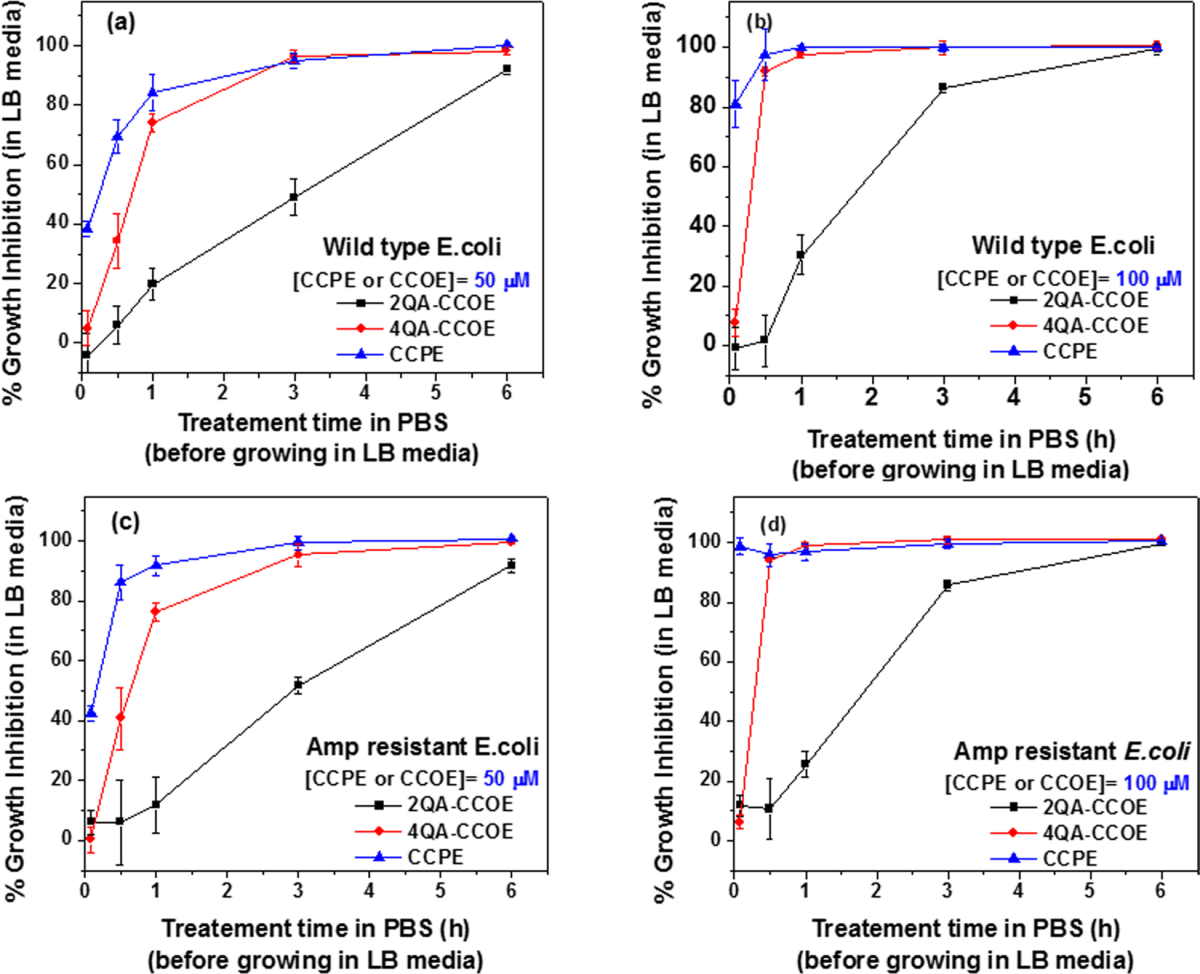
% growth inhibition of wild-type (**a,b**) and amp-resistant (**c,d**) *E. coli* in LB media for “treat (PBS), then grow (LB)” process. In this process, *E. coli* cells were treated first for 0–6 h in 50 μM (**a,c**) and 100 μM (**b,d**) of 2QA-CCOE, 4QA-CCOE, or CCPE in PBS. The cells were then transferred and allowed to grow in LB media for 3 h. Each data point represents the mean and standard deviation of 3 replicates.

In the “treat (PBS), then grow (LB)” process, wild-type and amp-resistant *E. coli* were treated first by mixing bacterial suspension in 5 mM PBS buffer with 2QA-CCOE, 4QA-CCOE, and CCPE. We varied the concentration of 2QA-CCOE, 4QA-CCOE, and CCPE between 0–100 μM in PBS in order to study the effect of concentration of conjugated molecules on antimicrobial properties. The cells were treated with conjugated molecules for 0–6 h at 37 °C. It is to be noted that similar *E. coli* treatment in PBS buffer with conjugated molecules has been demonstrated in several reports^25,31,40^. The utility of treating the bacteria in LB-free liquid (i.e. PBS here) is that the bacteria do not grow further during the treatment and that allow us to elucidate the sole effect of conjugated molecules on a specific population of bacteria. For measuring the percent of dead cells during CCOE/CCPE treatment, we performed a live/dead assay using flow cytometry (Fig. 3). In this assay, SYTO9 and PI stains were used, where SYTO9 dye is able to stain both damaged and intact cells, while PI stains damaged cells only^41,42^. The percent (%) of dead cells in *E. coli* suspension after incubation with conjugated molecules were measured from this assay and varied between 9–14%. In addition, the absorbance of the bacterial suspension in PBS buffer varied insignificantly (absorbance at 600 nm ~0.45–0. 55, see Supplementary Fig. S1) as a function of time of incubation in CCOEs or CCPE during this step. The UV/Vis supported the findings of live/dead assay (Fig. 3) and the fact that CCPE and CCOEs did not kill wild-type or amp-resistant *E. coli* predominantly in PBS buffer (during the treatment period). While cell lysis or death^20,43,44^ did not happen during this CCOE/CCPE treatment step in PBS buffer, there were prominent interactions between conjugated molecules and the bacterial outer cell envelope during this step. The effect of these interactions was evident in the subsequent step in which these bacterial cells were allowed to grow in LB media and percent growth inhibition was calculated (Fig. 4).

### Measurement of % growth inhibition

The CCOE/CCPE treated *E*. coli cells, when allowed to grow in LB media (“treat (PBS), then grow (LB)” process), the bacterial growth was efficiently inhibited (Fig. 4). The observed % growth Inhibition of *E. coli* (calculated using Eq. (1) as explained in Materials and Methods section) was consistently higher when *E. coli* was treated with CCPE in PBS before allowing it to grow in LB media (Fig. 4). Even when *E. coli* (both wild-type (Fig. 4a) and amp-resistant (Fig. 4c)) were treated with 50 μM CCPE for a few minutes (~3–5 min) in PBS, bacteria grew less than untreated bacteria and led to 40% growth inhibition. This indicated an instant action of CCPE on *E. coli* in PBS buffer. The % growth inhibition was 80% (Fig. 4b, wild-type) or above (Fig. 4d, amp-resistant) when CCPE concentration in treatment solution was increased from 50 (Fig. 4a,c) to 100 μM (Fig. 4b,d). 2QA-CCOE and 4QA-CCOE were also able to inhibit the growth of *E. coli*, but needed longer treatment to generate the inhibitory effect similar to CCPE. We observed 95% growth inhibition when *E. coli* cells were treated with 50 μM 4QA-CCOE or CCPE for 3 h. However, to achieve this level of growth inhibition with 2QA-CCOE, doubling the incubation time was required (6 h) (Fig. 4a,c). A higher concentration of conjugated molecules (100 μM, Fig. 4b,d) helped to reach ~99% growth inhibition faster (within ~3–5 min, 1 h and 6 h with CCPE, 4QA-CCOE and 2QA-CCOE, respectively for amp-resistant *E. coli* (Fig. 4d)). In addition, both CCOEs and CCPE were able to inhibit the growth of antibiotic-resistant bacteria to a similar extent as wild-type strains. The fact that the bacteria were not dying during the treatment step with CCOE/CCPE in PBS buffer, but not growing significantly when transferred to LB media evoked interest. The primary speculations were that it is either a straightforward effect of CCOE/CCPE attachment to bacteria which did not let the bacteria grow/divide further or an effect of PBS buffer during the treatment stage which negatively affected the metabolism of bacteria. Our control experiments showed that if the bacteria cells were kept in PBS buffer (without CCOE/CCPE) and later transferred to grow in LB media, the cells continued to grow in LB media. This suggests that possibly PBS buffer is not significantly affecting the metabolism. If something was decreasing the metabolism, it was likely the attachment and interaction of CCOE/CCPE with bacterial outer membrane. Zeta potential measurements (shown in a later section) will provide evidence of attachment of CCOEs/CCPE with bacterial cells.

Later, we measured the % growth inhibition following the other two treatment strategies mentioned earlier (i.e. (“treat (LB), then grow (LB)” process), (Supplementary Fig. S2), and, “treat and grow (LB)” process (Supplementary Fig. S3, Supplementary Table S2). It appeared that none of these two processes were effective in exhibiting growth inhibition (Supplementary Fig. S2, Supplementary Table S2). Through calculation using maximum CCPE adsorption by bacteria (Supplementary Fig. S4), we found that the number of CCPE chains available in both of these treatment systems were much higher than that needed to effectively coat all the bacterial cells present. For example, by using 100 μM CCPE in the treatment system, we offered 1.06×10^15^ chains of CCPE/mL which is ~5 times higher than what was needed for achieving the most efficient coating of the cells. This suggested that not having enough CCPE (to coat bacteria) was not the root cause for the poor growth inhibition in these two treatment processes. One thing in common between “treat (LB), then grow (LB)” process, and, “treat and grow (LB)” process was: in both cases, CCOE/CCPE chains were in LB media. We thus hypothesized that the poor antimicrobial activity of CCOEs and CCPEs in LB media was due to high ionic strength of the LB media (171 mM) which led to charge screening of both bacteria and cationic conjugated molecules and minimized their electrostatic interactions. The hypothesis was supported by the zeta potential reported for *E. coli* cells in solution with various ionic strengths (discussed in detail in supporting information). Based on all these observations for different modes of treatment and culture, “treat (PBS), then grow (LB)” process (Fig. 4) appeared to be the most effective mode to attain bacterial growth inhibition.

**Figure 5:**
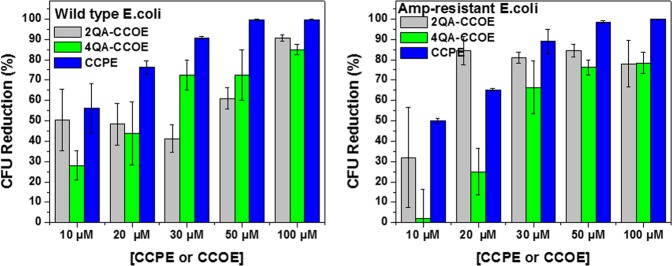
% CFU reduction of wild-type (left) and amp-resistant (right) *E. coli* grown on agar plates after 30 min treatment with 2QA-CCOE (light grey), 4QA-CCOE (green), and CCPE (blue) in PBS (2 step; “treat (PBS), then grow (LB)” process). The concentration of CCPE and CCOEs varied between 10 and100 μM. % CFU reduction values were calculated with respect to untreated cells (based on equation (2)). The data represents mean and standard deviation of 3 replicates for each treatment.

The growth inhibition data for *E. coli* (“treat (PBS), then grow (LB)” process) was further supported by colony-forming unit (CFU) reduction assays done on agar plates (Fig. 5 and Supplementary Fig. S5 (wild-type), S6 (amp-resistant)). Briefly, 5 μL of CCOEs/CCPE-treated bacterial suspension (after 10^4^ times dilution in 5 mM PBS) was spread over each agar plate and allowed to grow for 15 h (37 °C). % CFU reduction was calculated according to equation (2) as explained in Materials and Methods section. As the concentration of CCPE or 4QA-CCOE in the treatment system increased, the *E. coli* growth on agar plates decreased gradually (i.e. % CFU reduction increased) (Fig. 5 and Supplementary Figs. S5 and S6). CCPE-treated *E. coli* showed the highest % CFU reduction, irrespective of CCPE concentration. Using a treatment system containing only 30 μM CCPE, ~90% CFU reduction was achieved which reached up to ~100% when CCPE concentration was ≥50 μM (Fig. 5). This suggested that CCPE can prevent the growth of wild-type *E. coli* similar to ampicillin if CCPE concentration is ≥50 μM (Supplementary Fig. S7). On the other hand, using 30 μM 2QA-CCOE and 4QA-CCOE in a treatment system, ≤50% CFU reduction of wild-type *E. coli* (Fig. 5, left) was achieved. In the case of CCOEs, % CFU reduction approached to values close to that of CCPE at higher concentration of CCOEs. This CFU reduction data supported that CCPE based treatment is faster and more effective to inhibit the growth of *E. coli* than those based on CCOEs with lower concentration of the conjugated molecule. Also, 2QA-CCOE, unlike 4QA-CCOE and CCPE, led to anomalous growth and random values of CFU units rather than a gradual decrease (Fig. 5 and Supplementary Figs. S5 and S6) with the increase in 2QA-CCOE concentration. This could be attributed to the lower water solubility of 2QA-CCOE (~1 mg/mL) as compared to 4QA-CCOE, and CCPE (~20 mg/mL for both). The numbers of cationic quaternary amine groups for every 3 benzene rings along backbone were 2, 3 and 4 for 2QA-CCOE, CCPE and 4QA-CCOE, respectively. The poor hydrophilicity made it difficult for 2QA-CCOE to consistently interact with the net negatively charged outer cell envelope of *E. coli*. On the other hand, a large number (~57) of RUs^37^, being wired in a chain, with a suitable charge density made CCPE more favorable to attach to the *E. coli* surface and inhibit the growth of *E. coli* more efficiently.

### Zeta potential of CCOE/CCPE treated *E. coli*

**Figure 6:**
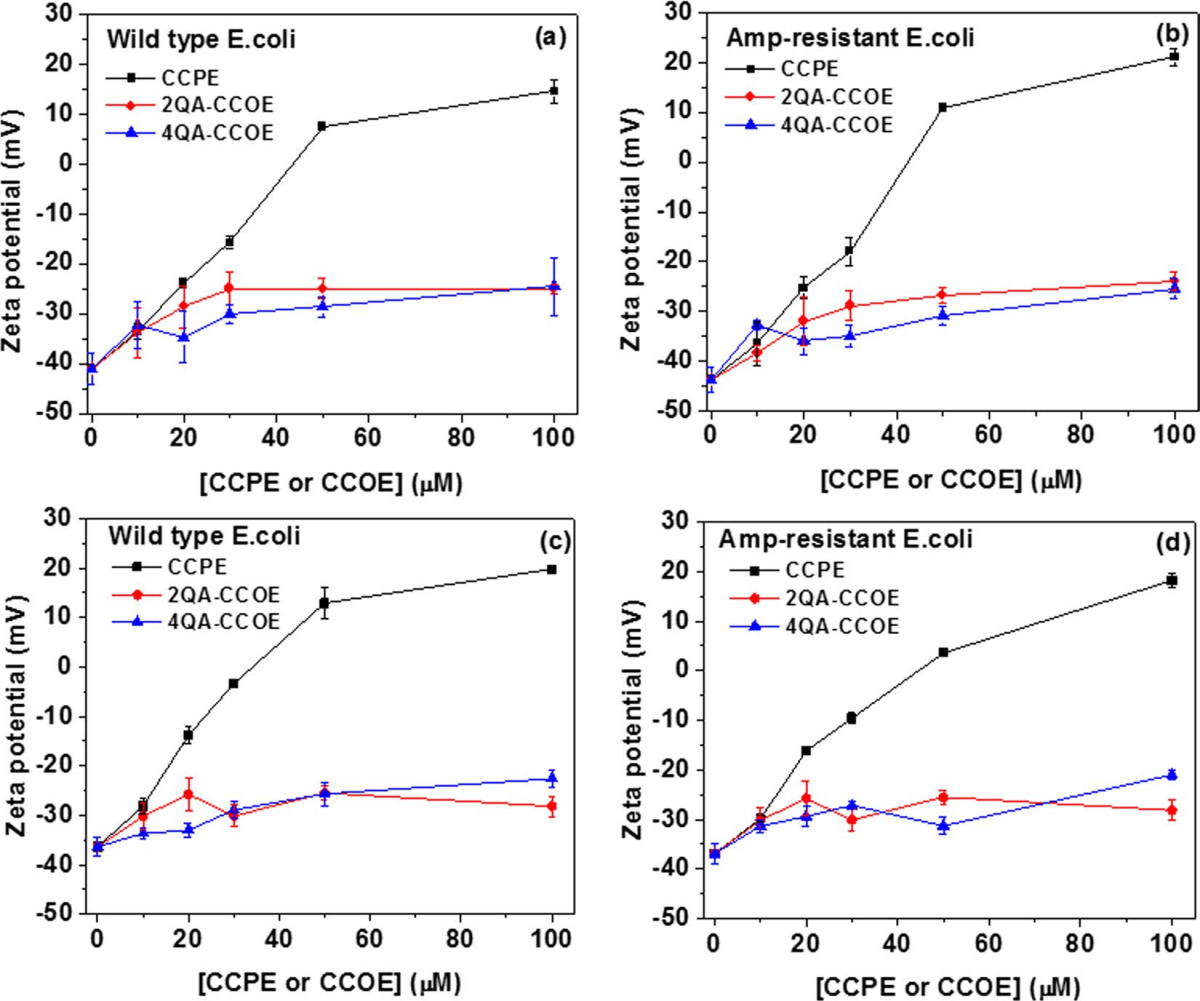
Zeta potential of wild-type (**a,c**) and amp-resistant (**b,d**) *E. coli* suspension after treatment with CCOE/CCPE for 1 h (**a,b**) and 6 h (**c,d**) in 5 mM PBS buffer. Each data point represents the mean and standard deviation of 2 replicates.

During the treatment process, the zeta potential also significantly changed as shown in Fig. 6. The untreated wild-type and amp-resistant *E. coli*  both were net negatively charged with zeta potential of ~−40 mV (Fig. 6) suggesting similar charged nature of  this specific *E. coli* strain after acquiring ampicillin resistance. Treatment with CCPE solution resulted in a large change in zeta potential (+15 mV (wild type, Fig. 6a) or +20 mV (amp-resistant, Fig. 6b)) of bacterial surface. Such a large surface charge reversal was expected based on high % CCPE adsorption on the bacteria surface (Supplementary Fig. S8). Interestingly, upon treatment with CCOEs, a much lower surface charge reversal of *E. coli* was observed (~−25 mV for both wild type and amp-resistant strains) at staining concentration ≥50 μM. Such a small change in zeta potential did not scale with the % mass adsorption, especially for 4QA-CCOE (about 65% mass adsorption of 4QA-CCOE (Supplementary Fig. S8d) was accompanied by a change in zeta potential from −37 mV to −27 mV only (Fig. 6)). We, therefore, believe that there are factors other than mass adsorption which controls the zeta potential (or surface charge) of *E. coli* treated with CCOE. Such a small increase in the zeta potential of bacteria treated with CCOEs was reported by others^6,21,29,32,33^ and attributed to the intercalation of CCOEs within the lipid bilayer of the outer membrane of *E. coli*. CCOEs with shorter chain length are more prone to membrane insertion^32^ and if the intercalation buries the CCOE chains and their cationic groups within lipid bilayer (rather than exposing the charges of CCOEs on the bacterial surface), the surface charge of bacteria may not change significantly despite CCOE adsorption.

The calculated backbone lengths of both 2QA-CCOE and 4QA-CCOE (~1.39 nm) were smaller than the thickness of the lipopolysaccharide layer (~2.8 nm^45^, where the length of the core oligosaccharide^6,45,46^ part is ~2.1 nm) of the outer cell membrane of *E. coli*. Lipid bilayer intercalation is thus more probable with CCOEs. On the other hand, the long-chain CCPEs (~57 RUs) may experience steric hindrance to penetrate into the lipid bilayer and peptidoglycan layer of *E. coli*^18,23^. Moreover, large charge reversals, similar to our CCPE coated bacteria, have been observed for cell surfaces coated with many other long-chain polyelectrolytes^47,48^. In fact, a similar reversal of surface charge was reported for poly(fluorene) based CCPE coated *E. coli*^30^. Whitten *et al*.^18,23^ proposed that the biocidal activity of CCPEs may originate not just from their lipid membrane perturbation activity, but also from their interactions with the charged functional groups of lipid bilayer exposed to cell surfaces (contributed by zwitterionic phospholipid head groups; charged groups of outer and inner core oligosaccharides, and, Lipid A)^5,49^. Based on these pieces of evidence, we propose that long-chain CCPE (~50 nm based on length of RU~0.889 nm) are predominantly forming a coating on the outer cell envelope of *E. coli*. However, the likelihood of minor CCPE chain intercalation (alongside coating as a major mechanism) cannot be ruled out since complete charge reversal was not achieved.

Some of the already established models support our prediction of binding interactions and antimicrobial mechanisms^18,50^. Coating of bacterial outer cell envelope resembles the carpet model^50,51^ since the antimicrobial molecules, when following this model, orient themselves parallel to the membrane surface and cover the bacterial surface, like a carpet (instead of inserting into the lipid membrane). On the other hand, CCOE intercalation can be explained by toroidal pore model^18^ based on which the membrane bound antimicrobial molecules insert into the lipid bilayer and force the outer leaflet to bend continuously to fuse with the inner leaflet of bacterial membrane^18^.

We also examined the effect of incubation time on the zeta potential of treated bacterial cells. A longer treatment time (6 h) did not change the zeta potential of treated bacteria much (Fig. 6(c,d)). Using 100 μM CCPE, ~99% growth inhibition of amp-resistant *E. coli* was achieved within 1 h (Fig. 4) which reached to 100% in the next 5 h. Additionally, the change in % mass adsorption (98% (1 h, Supplementary Fig. S8b); 98% (6 h, Supplementary Fig. S8d)) and zeta potential (+20 mV (1 h, Fig. 6b); +18 mV (6 h, Fig. 6d)) were minor. The results suggested that the adsorption of CCPE by bacteria was almost complete within 1 h of treatment and sufficient to achieve high % growth inhibition. Interestingly, the zeta potential of 2QA-CCOE treated amp-resistant bacteria reached a plateau by 1 h (~−25 mV after 1 h (Fig. 6b); −28 mV after 6 h (Fig. 6d)), but the bacteria needed the full 6 h-treatment to achieve 98% growth inhibition (growth inhibition was 25% after 1 h treatment (Fig. 4d)). This suggested that growth inhibition of 2QA-CCOE was a time-dependent process. Rapid intercalation (within 100 s, based on epifluorescence micrograph experiments) of CCOEs within the bacterial membrane was reported by Hinks *et al*.^32^ for oligo(phenylenevinylene) with 4 RUs and 4 quaternary amine groups. However, the timescales of their experimental (100 s) and simultaneous molecular dynamics simulation (200 ns) studies on membrane perturbation of Gram-negative bacteria were very small and an understanding of membrane perturbation over large time scale is required. The reason is: to achieve 99% growth inhibition using 2QA-CCOE, cells had to be treated with 2QA-CCOE for a longer time (6 h), but within the first 1 h of this treatment time, adsorption of conjugated molecules by bacteria was complete. These results strongly suggested that after 1 h treatment, the already-attached CCOEs took part in dynamic membrane perturbation processes which continued for the next 5 h of treatment. This led to time-dependent growth inhibition by CCOE treatment. Furthermore, there was no significant difference in the % mass adsorption of CCOE/CCPE on wild-type (Supplementary Fig. S8a,c) and amp-resistant (Supplementary Fig. S8b,d) *E. coli* and the resulting zeta potential (Fig. 6a,c vs 6b,d). These again supported that any specific type of conjugated molecule interacts with wild-type and amp-resistant *E. coli* (used in this study) in a similar manner.

### Morphological changes upon treatment with CCOEs/CCPE

**Figure 7:**
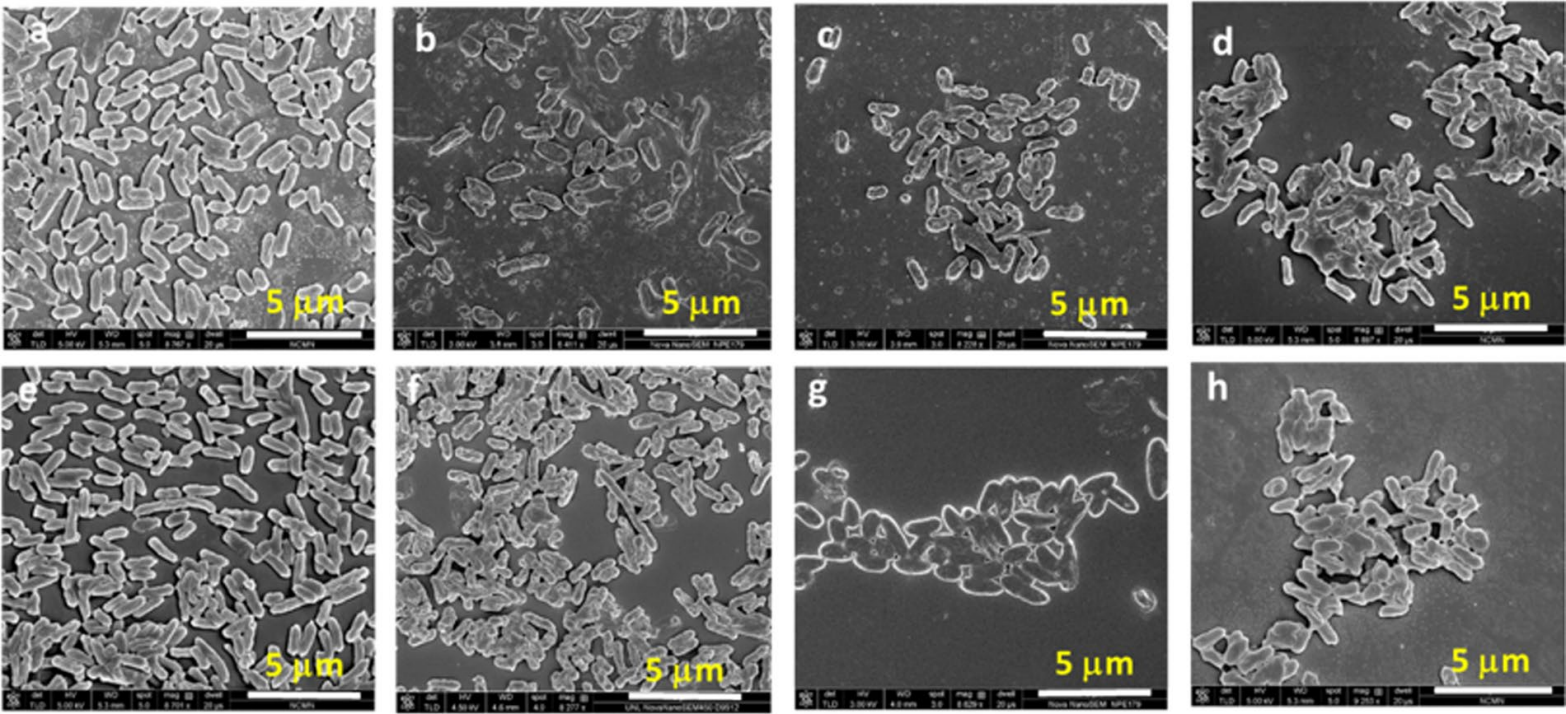
SEM images of wild type (top panel) and amp-resistant (bottom panel) *E. coli* without treatment (**a,e**); treated with 30 µM 2QA-CCOE (**b,f**), 4QA-CCOE (**c,g**), and CCPE (**d,h**).

Fluorescence microscopy images of green fluorescent protein (GFP)-incorporated amp-resistant *E. coli* suspension before and after treatment with 30 µM and 100 µM CCOEs/CCPE were captured (Supplementary Fig. S9). While CCOE-treatment did not change the size of *E*. *coli* aggregates (Supplementary Fig. S9a,b,c,e,f) and the cells were observed to spread out across the frame, bacteria started to aggregate significantly upon interaction with CCPE (Supplementary Fig. S9d,g). The size of aggregates (~15–20 μM) in CCPE (30 μM) treated *E*. *coli* suspension found from fluorescence microscopy image (Supplementary Fig. S9d) was similar to what obtained from SEM image (Fig. 7h). While interpreting the SEM data (Figure 7), we have to keep in mind that we are looking at 3D aggregates as a 2D image. Even though these are 2D images, the contrast can help us to understand the height wise growth of the aggregation. For untreated (Fig. 7a,e) and 30 μM of 2QA-CCOE-treated *E. coli* cells (Fig. 7b,f), the cells were lying flat like a single layer and spread out throughout the frame. When the cells were treated with 30 μM CCPE, the net negatively charged cells started to stick with each other in a three-dimensional manner due to electrostatic complexation assisted by cationic CCPE (Fig. 7d,h). In this case, the aggregates grew significantly along the third dimension, i.e. perpendicular to the image plane as evident from the image contrast (Fig. 7d,h). When the cells were treated with 4QA-CCOE (30 μM, Fig. 7c,g), cells were aggregated, but the height wise growth was less than the cells treated with CCPE. Cationic CCPE chains, when coated the negatively charged *E. coli* cells, can attract other uncoated, negatively charged bacteria cells around and form larger aggregates (Fig. 7d,h). Intercalated CCOEs, on the other hand, are likely to conceal the positive charges of CCOEs and minimize the tendency to form bacterial aggregates (Fig. 7b,c,f,g).

**Figure 8:**
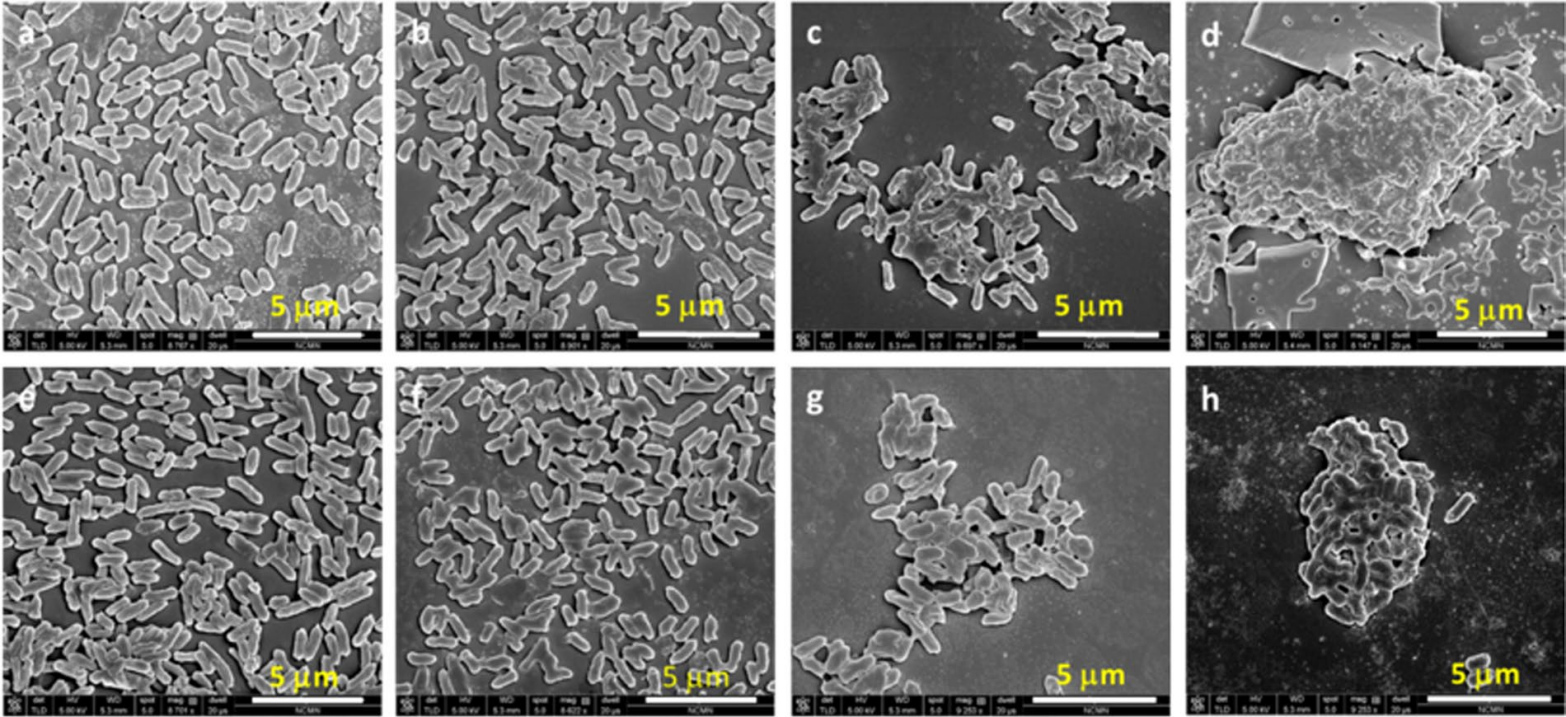
SEM images of wild type *E. coli* (top panel) and amp-resistant *E. coli* (bottom panel), without treatment (**a,e**); treated with 10 µM (**b,f**), 30 µM (**c,g**), and 100 µM CCPE (**d,h**).

Aggregates became larger and clearly grew in three dimensions when the concentration of CCPE in treatment solution increased from 0 μM to 100 μM (Fig. 8a–d for wild-type, 8e-h for amp-resistant *E. coli*) and seemed to be covered by CCPE molecules. No obvious cell wall rupture or cell lysis was predicted especially for CCOEs since the single cells in both treated (Fig. 7b,c,f,g) and untreated (Fig. 7a,e) *E. coli* suspensions looked smooth visually. Due to large aggregate formation, the individual cells could not be seen in the case of CCPE treated *E*. coli which made it difficult to comment about cell wall rupture/lysis based on SEM images. However, no GFP release from treated cells (Supplementary Fig. S9), low amount of dead cells based on live/dead assay (Fig. 3), and unchanged absorbance of treated bacteria (Supplementary Fig. S1) confirm no cell wall rupture/lysis upon treatment with CCPE. These results indicated that phenylene based CCOEs and CCPEs, unlike, phenylene ethynylene based ones^18,23^ (showing bacteriolytic activity), acted on the bacterial outer cell membrane more gently, but inhibited further growth. Having said that, the mild alterations at the single-cell level (non-bacteriolytic) could not be differentiated based on the SEM images (due to strong aggregation after treatment). But it is highly likely that mechanical changes are happening to some extent on bacterial outer cell envelope due to these treatments (currently under investigation). Finally, no significant difference in morphology between untreated wild-type (Fig. 7a) and amp-resistant (Fig. 7e) *E. coli* was observed and so was observed for treated ones (Fig. 7). This was consistent with the indifferent growth inhibition and zeta potential of these two strains.

### Conclusions

Through synthesizing phenylene based model cationic conjugated oligo- (2QA-CCOE, 4QA-CCOE) and polyelectrolytes (CCPE), we studied the biophysical changes on the outer cell envelopes of wild-type and amp-resistant *E. coli* strains upon interactions with these conjugated molecules. These conjugated molecules inhibited the growth of both wild-type and amp-resistant *E. coli* to a similar extent. About 99% growth inhibition was achieved if amp-resistant *E. coli* was treated for ~3–5 min, 1 h, and 6 h in 100 μM of CCPE, 4QA-CCOE, and 2QA-CCOE solutions, respectively. This indicated CCPE was a faster growth inhibitor of bacteria compared to oligomeric CCOEs. The better inhibitory activity of CCPE could be attributed to an optimum balance of side-chain hydrophilicity and backbone hydrophobicity, good water solubility as well as large chain length which made charge interaction of CCPE with net negatively charged bacteria more facile. The large charge reversal of bacteria upon treatment with CCPE suggested that bacterial cells were coated with CCPE chains. On the other hand, the low surface charge reversal suggested that CCOEs were intercalating within the lipid bilayer of the outer membrane of *E. coli*.

## Materials and Methods

### Bacteria culture

To make liquid growth media for *E. coli*, solid LB (Miller, AMRESCO) (25 g) was dissolved in DI water (1 L); while to make agar plates, solid LB (25 g) and agar powder (15 g) were dissolved in DI water (1 L). In both cases, the liquid LB media was autoclaved. Ampicillin (100 μg/mL) was then added to the media as needed. The liquid media was transferred to petri dishes to obtain solid agar plates. The agar plates were stored in the cold room (4 °C) till use. For each experiment, the *E. coli* strains stored in 15% glycerol were streaked on LB agar plates. The plates were incubated overnight at 37 °C. A single colony was then transferred into 4 mL of liquid LB media in 14 mL BD Falcon round-bottom culture tubes and incubated overnight at 37 °C in a shaker-incubator (250 rpm). Overnight grown cells were harvested by centrifugation (4700 rpm) for 15 min and the pellets were washed twice in 4 mL of 5 mM PBS. The cells were resuspended in 5 mM PBS and the optical density of resuspended cells measured at 600 nm (OD_600_) was then adjusted as needed using Genesys 10 S UV-Vis spectrophotometer (Thermo Fisher Scientific, Waltham, MA).

### Live/dead assay

Live/dead assay of wild-type *E. coli* was done using LIVE/DEAD BacLight bacterial viability and counting kit (Thermo Fisher Scientific, Waltham, MA) and samples prepared as described in the protocol^52^. Briefly, wild-type *E. coli* cells were grown for 8 h and OD_600_ was adjusted to 0.2 in 5 mM PBS. Cells were treated with CCOE/CCPE (10–50 μM) and incubated at 37 °C, 250 rpm for 1 h. Treated cells were harvested; centrifuged at 10000 × g for 3 minutes; pellets were washed twice with 0.85 wt % NaCl in water and resuspended in 1 mL of 0.85 wt% NaCl solution. Samples were then diluted in two steps: In the first step, the cells were diluted with appropriate volume of 0.85 wt% NaCl solution to achieve OD_600_ of bacterial suspension as 0.125; this cell suspension was then further diluted to obtain 1 million cells/mL by adding 10 µL of cell suspension (OD_600_ = 0.125) to 987 µL of 0.85% NaCl solution. Finally, 3 µL of mixed SYTO9 and PI (1:1 v/v) was added to the cell suspension and used for flow cytometry. Each treatment had 2 replicates. BD FACSAria II (Franklin Lakes, NJ) at Nebraska Center for Biotechnology at University of Nebraska-Lincoln was then used for flow cytometry studies. BD FACSDiva software was used to analyze the flow cytometry data and obtain % of damaged cells.

### Absorbance measurement upon CCOE/CCPE treatment

CCOE/CCPE and cell suspension were added together in 5 mM PBS ([CCOE/CCPE]_after adding to PBS_ = 0–100 μM (based on RUs); absorbance of *E. coli* at 600 nm ~0.5 (initially); final volume = 250 μL). The suspension was incubated for 0–6 h (37 °C, 250 rpm). The change in absorbance at 600 nm was measured using i3xplate reader (Molecular Devices, San Jose, CA). The study was performed for both wild-type (DH10B) and amp-resistant *E. coli* (SSBIO002) (as shown in Supplementary Fig. S1)). This procedure was followed to replicate the data three times.

### Measurement of percent (%) growth inhibition

The growth inhibition was studied in three ways: “treat and grow (LB)” (*1-step-process*); “treat (PBS), then grow (LB)” (*2-step-process*); and “treat (LB), then grow (LB)” (*2-step-process*). For the *1-step process*, the CCOE/CCPE and cell suspension were added together in LB media ([CCOE/CCPE]_after adding to LB_ = 0–100 μM (based on RUs); OD_600, initial_ = 0.2; final volume = 250 μL) and the cells were allowed to grow here for 0–6 h (37 °C, 250 rpm). For the 2-step processes, the procedure was similar to what described in the literature^22^. At first, the *E. coli* strains (OD_600, initial_ = 0.2) were treated with CCOE/CCPE for 0–6 h (37 °C, 250 rpm) in PBS (for “treat (PBS), then grow (LB)” process) or LB (for “treat (LB), then grow (LB)” process). After that, 50 μL of this treated cell suspension was transferred to 200 μL of LB media in black 96-well plates and allowed to grow for 3 h (37 °C, 250 rpm). The percent (%) growth inhibition of *E. coli* in LB media was calculated using the Eq. (1):

1 $$\begin{array}{c}\,\,\,\,\, \% \,{\rm{Growth}}\,{\rm{Inhibition}}\\ \,=\,\frac{{(Abs)}_{{\rm{untreated}}E.coli{\rm{after}}{\rm{growing}}{\rm{in}}{\rm{LB}}}-{(Abs)}_{\text{CCOE}/\text{CCPE}{\rm{treated}}E.coli{\rm{after}}{\rm{growing}}{\rm{in}}{\rm{LB}}}}{{(Abs)}_{{\rm{untreated}}E.coli{\rm{after}}{\rm{growing}}{\rm{in}}{\rm{LB}}}}\times 100 \% \end{array}$$

Each absorbance value was background subtracted where the background was the absorbance of the sample at time = 0 (i.e. just after adding to LB media).

### Colony-forming unit (CFU) reduction assay

The protocol described in the literature was used for CFU reduction studies^25^. Briefly, the bacterial strains grown in liquid LB media were used and OD_600_ was adjusted to 0.2 by dilution with 5 mM PBS buffer. 180 μL of this *E. coli* suspension was transferred to black 96-well plates to which appropriate volume of a type of conjugated molecule (i.e., 2QA-CCOE, 4QA-CCOE, or CCPE) was also added. The final concentration of CCOEs or CCPE in this pretreatment step varied between 0 and 100 μM (based on RUs). The treated bacterial cells were diluted 10^4^ times using 5 mM PBS. 5 μL of this diluted bacterial suspension was then spread on top of the agar plates (3 replicates for each treatment) and allowed to grow in dark for 15 h at 37 °C. The number of CFUs was then counted and CFU reduction (%) was calculated using the Eq. (2):

2 $$\begin{array}{c}\,\,{\rm{ \% }}\,{\rm{C}}{\rm{F}}{\rm{U}}\,{\rm{r}}{\rm{e}}{\rm{d}}{\rm{u}}{\rm{c}}{\rm{t}}{\rm{i}}{\rm{o}}{\rm{n}}\\ \,=\,\frac{{(CFU)}_{{\rm{u}}{\rm{n}}{\rm{t}}{\rm{r}}{\rm{e}}{\rm{a}}{\rm{t}}{\rm{e}}{\rm{d}}E.coli{\rm{a}}{\rm{f}}{\rm{t}}{\rm{e}}{\rm{r}}15{\rm{h}}{\rm{o}}{\rm{n}}{\rm{a}}{\rm{g}}{\rm{a}}{\rm{r}}{\rm{p}}{\rm{l}}{\rm{a}}{\rm{t}}{\rm{e}}}\,-{(CFU)}_{{\rm{C}}{\rm{C}}{\rm{O}}{\rm{E}}/{\rm{C}}{\rm{C}}{\rm{P}}{\rm{E}}{\rm{t}}{\rm{r}}{\rm{e}}{\rm{a}}{\rm{t}}{\rm{e}}{\rm{d}}E.coli{\rm{a}}{\rm{f}}{\rm{t}}{\rm{e}}{\rm{r}}15{\rm{h}}{\rm{o}}{\rm{n}}{\rm{a}}{\rm{g}}{\rm{a}}{\rm{r}}{\rm{p}}{\rm{l}}{\rm{a}}{\rm{t}}{\rm{e}}}}{{(CFU)}_{{\rm{u}}{\rm{n}}{\rm{t}}{\rm{r}}{\rm{e}}{\rm{a}}{\rm{t}}{\rm{e}}{\rm{d}}E.coli{\rm{a}}{\rm{f}}{\rm{t}}{\rm{e}}{\rm{r}}15{\rm{h}}{\rm{o}}{\rm{n}}{\rm{a}}{\rm{g}}{\rm{a}}{\rm{r}}{\rm{p}}{\rm{l}}{\rm{a}}{\rm{t}}{\rm{e}}}}\times 100{\rm{ \% }}\end{array}$$

Plain LB and LB-supplemented with ampicillin (100 μg/mL) were used to grow the wild-type and amp-resistant strains, respectively. The CFUs for untreated amp-resistant *E. coli* grown on an agar plate with and without ampicillin were almost similar (Supplementary Fig. S10). This confirmed the effectiveness of colony selection process and growth of amp-resistant *E. coli* only (i.e. no growth of wild-type *E. coli*). Also, the wild-type and amp-resistant *E. coli* were grown on agar plates made of LB supplemented with ampicillin (Supplementary Fig. S11). While the amp-resistant *E. coli* grew on agar plates, there was no colony formation for wild-type *E. coli*. This confirmed that just amp-resistant *E. coli* was able to grow.

### Zeta potential measurement

Both wild-type and amp-resistant *E. coli* samples were treated with CCOEs or CCPE (as mentioned in prior sections) after which the cells were harvested by centrifugation (4700 rpm, 15 min). The pellets were washed twice with DI water (2 ml per wash) and then resuspended in 2 mL DI water for subsequent zeta potential measurement using Zeta PALS Zeta Potential Analyzer (Brookhaven Holtsville, NY).

### Scanning electron microscope (SEM) imaging

For the investigation of the morphology of untreated and CCPE/CCOE treated *E. coli*, scanning electron microscope (SEM; Nova NanoSEM450, FEI, Hillsboro, OR) was performed at Nebraska Center for Materials and Nanoscience. 900 μL of bacterial suspension (OD_600_ = 0.2) and different concentrations of CCPE or CCOEs (0–100 μM) were mixed and incubated for 1 h in culture tubes (37 °C, 250 rpm). The treated cells were harvested by centrifugation (4700 rpm, 15 min) and pellets were washed twice (with 1 mL of DI water per wash) and resuspended in 1 mL DI water. 3 μL of this suspension was added on top of a silicon wafer (0.5 cm × 0.5 cm) and allowed to dry at room temperature for 1 h. Dried samples were fixed with 0.5 vol% glutaraldehyde in 5 mM PBS buffer and kept at room temperature for another hour. This was followed by a second fixing using 1 vol% glutaraldehyde in 5 mM PBS and samples were kept at room temperature for 4 h. Fixed cells were washed thrice with DI water and dehydrated sequentially using 20, 30, 50, 70, 90 and 100 vol% of ethanol in water. Finally, the specimens were coated with gold prior to SEM measurement.

## Supplementary information

Supplementary Information.

## Data Availability

All data generated and analyzed in this study are included in main text or Additional Information.
