# Supplementary material for: Mechanistic Understanding of the Interactions of Cationic Conjugated Oligo- and Polyelectrolytes with Wild-type and Ampicillin-resistant Escherichia coli
Source: Sci Rep. 2019 Dec 31;9:20411. doi: 10.1038/s41598-019-56946-2 (PMC6938524; doi:10.1038/s41598-019-56946-2)
Supplement: Supplementary file 1 — Supplementary Information. [file 41598_2019_56946_MOESM1_ESM.pdf]

Supporting Information

Mechanistic Understanding of the Interactions of Cationic Conjugated Oligo- and Polyelectrolytes with Wild-type and Ampicillin-resistant *Escherichia coli*

Ehsan Zamani,[1] Shyambo Chatterjee,[1] Taity Changa,[1] Cheryl Immethun,[1] Anandakumar Sarella,[2] Rajib Saha,[1] Shudipto Konika Dishari[1,]*

[1,]* Department of Chemical and Biomolecular Engineering, University of Nebraska-Lincoln, Lincoln, Nebraska 68588, United States

[2] Nebraska Center for Materials and Nanoscience, Voelte-Keegan Nanoscience Research Center, University of Nebraska-Lincoln, Lincoln, NE 68588-0298, United States

*Corresponding author's email: sdishari2@unl.edu; Phone: 402-472-7537.

Experimental Section.

Materials and methods for synthesis and characterization of CCOEs and CCPE. Neutral conjugated polymer, Poly{[2,5-bis(2-(*N*,*N*-diethylamino)ethoxy)-1,4-phenylene]-*alt*-1,4-phenylene} (**P_n**) with M_n of 8.8 kg/mol (PPP standard) and PDI of 2.05 was purchased from Sigma-Aldrich (678066, St. Louis, MO). 2,5-dibromohydroquinone (Sigma-Aldrich), used for Suzuki coupling, was recrystallized before use from hot pentane. Tetrahydrofuran (THF) (Acros Organics, NJ), used in the quaternization reaction, was distilled from Na/benzophenone under inert atmosphere unless otherwise noted. All other reagents and solvents were obtained from commercial suppliers (Sigma-Aldrich (St.Louis, MO)); TCI America (Portland, OR); Acros organics (NJ)) and used without further purification unless otherwise stated. Air and moisture-sensitive reactions were conducted using oven-dried glassware and standard technique under an inert atmosphere of dry argon. ^{1}H NMR and ^{13}C NMR were recorded on Bruker-DRX-Avance (300, 400, and 600 MHz). Samples were dissolved in deuterated chloroform ($CDCl_3$) and dimethylsulfoxide-deuterated water (DMSO-D6: D2O (2:1)) to collect ^{1}H and ^{13}C NMR spectra. Chemical shifts (δ) were given in ppm relative to the internal standard. High-resolution mass spectra (HRMS) were acquired at the Nebraska Center for Mass Spectroscopy (NCMN, University of Nebraska). Samples of 2QA-CCOE, 4QA-CCOE, and CCPE were sent to Galbraith Laboratories, Inc. (Knoxville, TN) to perform elemental analysis.

Materials for bacteria culture. Luria Bertani (LB) broth, Miller (AMRESCO, Solon, OH) was used as culture media. Bacteriological grade agar (Lab Scientific, Highlands, NJ) and ampicillin (Research Products International, Mount Prospect, IL) were purchased and used as received. 10X Phosphate buffer (Fisher Scientific, Hampton, NH) was purchased and diluted to 5 mM using DI water for further use. Ethanol (Decon Labs, King of Prussia, PA) and glutaraldehyde (Sigma-Aldrich, St. Louis, MO) were procured for scanning electron microscopy (SEM) measurements. Black 96-well plates (Greiner Bio-One µClear™ Bottom 96-Well Polystyrene Microplates, flat bottom, chimney well) were purchased from Fisher Scientific (Hampton, NH) to incubate *E. coli* with cationic conjugated molecules (2QA-CCOE, 4QA-CCOE, CCPE) and measure absorbance of *E. coli* suspension. Glass slides and silicon wafers (C04004) were purchased from Home Science Tools (Billings, MT) and Waferpro (San Jose, CA), respectively.

Plasmid assembly in bacterial strains. *E. coli* (DH10B, wild-type, (NEB #C3020K)) was purchased from New England Biolabs (Ipswich, MA). *E. coli* MG1655 (CGSC #6300) was purchased from the *E. coli* Genetic Stock Center at Yale University (New Haven CT). *E. coli* MG1655's genomic DNA (gDNA) was extracted and P_{Lac}, which was used as a promoter to express green fluorescent protein (*gfpuv*), was copied from the gDNA using PCR. The plasmid pBb7a-GFP[1] was purchased from Addgene (Watertown, MA) and the genetic parts *pBBR1* replicon, amp^R and *gfpuv* were copied from the plasmid using PCR. The sequences are listed in the Table S1 in the supplementary information. The plasmid pSSBIO002 (*pBBR1* replicon, amp^R) was assembled *via* DNA ligation;[2] while the Hot Fusion method[3] was used for assembling plasmid pSSBIO101 (*pBBR1* replicon, amp^R, P_{Lac}, *gfpuv*). *E. coli* DH10B, transformed with pSSBIO101, produces green fluorescent protein; while both pSSBIO002 and pSSBIO101 confer ampicillin resistance. The strains (DH10B, SSBIO002, SSBIO101) were stored (in 15% glycerol) in -80 °C freezer and were further used as needed to grow wild-type and amp-resistant *E. coli.*

CCOE/CCPE adsorption on bacterial outer membrane. After pretreatment with CCOE/CCPE at varied concentrations (30-100 μM), *E. coli* suspension was centrifuged to collect the supernatant (having CCPE/CCOE which was not adsorbed). The fluorescence intensities of CCOE /CCPE solution (without bacteria) and the supernatant were obtained to calculate % mass adsorption of CCOE/CCPE using the equation (3):

$$\%\ \text{Adsorption of CCOE or CCPE} = \frac{(\text{PL intensity})_{\substack{CCOE\ or\ CCPE\ in\ treatment\ solution \\ without\ bacteria}} - (\text{PL intensity})_{\substack{CCOE\ or\ CCPE\ in\ supernatant \\ collected\ after\ treatment}}}{(\text{PL intensity})_{\substack{CCOE\ or\ CCPE\ in\ treatment\ solution \\ without\ bacteria}}} \times 100\% \quad \text{........(3)}$$

Fluorescence microscopy imaging. A single colony of amp-resistant *E. coli* expressing green fluorescent protein (SSBIO101) was cultured overnight in 4 mL of LB media supplemented with ampicillin in 14 mL BD Falcon round-bottom culture tubes on a shaker-incubator (250 rpm) at 37 °C. Cells were harvested the next day by centrifugation at 4700 rpm for 15 min. The supernatant was discarded and the pellets were resuspended in 5mM PBS followed by washing (twice) in 4 mL of PBS (per wash). Finally, OD_{600} of resuspended cells was adjusted to 1 using the same PBS solution. Aqueous solution of 2QA-CCOE, 4QA-CCOE or CCPE was then added to 900 µL of *E. coli* suspension (OD_{600} = 1) in culture tubes to yield a total volume of 1 mL and a final concentration of 0 and 30 µM of CCPE or CCOEs. Treated cells were incubated for 1 h in the dark (37 ºC, 250 rpm). After incubation, cells were harvested by centrifugation and washed again in 5 mM PBS as mentioned before and resuspended in 1 mL of the same buffer. To prepare the samples for fluorescence microscopy, about 5-10 µL of this cell suspension was transferred

onto a clean glass slide and cover slip was applied. The samples were then viewed under EVOS FL Auto fluorescence microscope (Life Technologies, Carlsbad, CA) using GFP filter (excitation at 488 nm) and 60X magnifications at Nebraska Center for Biotechnology. The captured images were processed using ImageJ software (an open resource from NIH).

Table S1. List of genetic parts used in this research.

Part name	Type and source	DNA sequence
amp^{R}	Gene[1]	Atgagtattcaacatttccgtgtcgcccttattcccttttttgcggcattttgccttcctgtttttgctcacccagaaa<br>cgctggtgaaagtaaaagatgctgaagatcagttgggtgcacgagtgggttacatcgaactggatctcaaca<br>gcggtaagatccttgagagttttcgccccgaagaacgttttccaatgatgagcacttttaaagttctgctatgtgg<br>cgcggtattatcccgtattgacgccgggcaagagcaactcggtcgccgcatacactattctcagaatgacttg<br>gttgagtactcaccagtcacagaaaagcatcttacggatggcatgacagtaagagaattatgcagtgctgcca<br>taaccatgagtgataacactgcggccaacttacttctgacaacgatcggaggaccgaaggagctaaccgcttt<br>tttgcacaacatgggggatcatgtaactcgccttgatcgttgggaaccggagcataccaaacgacgagcgtg<br>acaccacgatgcctgtagcaatggcaacaacgttgcgcaaactattaactggcgaactacttactctagcttcc<br>cggcaacaattaatagactggatggaggcggataaagttgcaggaccacttctgcgctcggcccttccggct<br>ggctggtttattgctgataaatctggagccggtgagcgtgggtctcgcggtatcattgcagcactggggccag<br>atggtaagccctcccgtatcgtagttatctacacgacggggagtcaggcaactatggatgaacgaaatagac<br>agatcgctgagataggtgcctcactgattaagcattggtaa
	Promoter	cgcggaacccctatttgtttatttttctaaatacattcaaatatgtatccgctcatgagacaataaccctgataaatg<br>cttcaataatattgaaaaaggaagagt
gfpuv	Gene[1]	atgagtaaaggagaagaacttttcactggagttgtcccaattcttgttgaattagatggtgatgttaatgggcaca<br>aattttctgtcagtggagagggtgaaggtgatgcaacatacggaaaacttacccttaaatttatttgcactactg<br>gaaaactacctgttccgtggccaacacttgtcactactttctcttatggtgttcaatgcttttcccgttatccggatc<br>acatgaaacggcatgactttttcaagagtgccatgcccgaaggttatgtacaggaacgcactatatctttcaaa<br>gatgacgggaactacaagacgcgtgctgaagtcaagtttgaaggtgatacccttgttaatcgtatcgagttaaa<br>aggtattgattttaaagaagatggaaacattctcggacacaaactggagtacaactataactcacacaatgtata<br>catcacggcagacaaacaaaagaatggaatcaaagctaacttcaaaattcgccacaacattgaagatggctc<br>cgttcaactagcagaccattatcaacaaaatactccaattggcgatggccctgtccttttaccagacaaccatta<br>cctgtccacacaatctgccctttcgaaagatcccaacgaaaagcgtgaccacatggtccttcttgagtttgtaac<br>tgctgctgggattacacatggcatggatgagctctacaaataa
	5 ′ UTR	aattgtgagcggataacaatttcatttcagaattcaaaagatcttttaagaaggagatatacat

PLac	Promoter<br>*E. coli* MG1655<br>GenBank: CP027060.1: 2639763 to 2639873[a]	ggcagtgagcgcaacgcaattaatgtgagttagctcactcattaggcaccccaggctttacactttatgcttcc<br>ggctcgtatgttgtgtggaattgtgagcggataacaa
pBBR1 oriV	Origin of replication[4]	gcggccaccggctggctcgcttcgctcggcccgtggacaaccctgctggacaagctgatggacaggctgc<br>gcctgcccacgagcttgaccacagggattgcccaccggctacccagccttcgaccacatacccaccggctc<br>caactgcgcggcctgcggccttgccccatcaatttttttaattttctctggggaaaagcctccggcctgcggcct<br>gcgcgcttcgcttgccggttggacaccaagtggaaggcgggtcaaggctcgcgcagcgaccgcgcagcg<br>gcttggccttgacgcgcctggaacgacccaagcctatgcgagtgggggcagtcgaaggcgaagcccgcc<br>cgcctgcccccgagcctcacggcggcgagtgcgggggttccaagggggcagcgccaccttgggcaag<br>gccgaaggccgcgcagtcgatcaacaagccccggaggggccactttttgccggagggggagccgcgcc<br>gaaggcgtgggggaaccccgcaggggtgcccttctttgggcaccaaagaactagatatagggcgaaatgc<br>gaaagacttaaaaatcaacaacttaaaaaagggggggtacgcaacagctcattgcggcaccccccgcaatag<br>ctcattgcgtaggttaaagaaaatctgtaattgactgccacttttacgcaacgcataattgttgtcgcgctgccga<br>aaagttgcagctgattgcgcatggtgccgcaaccgtgcggcaccctaccgcatggagataagc
pBBR1 replication protein	Gene[4]	atggccacgcagtccagagaaatcggcattcaagccaagaacaagcccggtcactgggtgcaaacggaac<br>gcaaagcgcatgaggcgtgggccgggcttattgcgaggaaacccacggcggcaatgctgctgcatcacct<br>cgtggcgcagatgggccaccagaacgccgtggtggtcagccagaagacactttccaagctcatcggacgtt<br>ctttgcggacggtccaatacgcagtcaaggacttggtggccgagcgctggatctccgtcgtgaagctcaacg<br>gccccggcaccgtgtcggcctacgtggtcaatgaccgcgtggcgtggggccagccccgcgaccagttgcg<br>cctgtcggtgttcagtgccgccgtggtggttgatcacgacgaccaggacgaatcgctgttggggcatggcga<br>cctgcgccgcatcccgaccctgtatccgggcgagcagcaactaccgaccggccccggcgaggagccgcc<br>cagccagcccggcattccgggcatggaaccagacctgccagccttgaccgaaacggaggaatgggaacg<br>gcgcgggcagcagcgcctgccgatgcccgatgagccgtgttttctggacgatggcgagccgttggagccg<br>ccgacacgggtcacgctgccgcgccggtag

[a] https://www.ncbi.nlm.nih.gov/nucleotide/CP027060.1

Synthesis of CCOEs and CCPE.

Detailed synthesis of 2QA-CCOE.

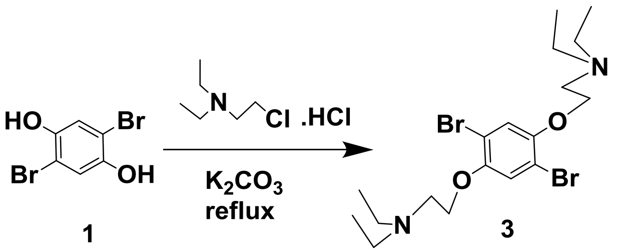

2,5-Bis(3-[N, N-diethylamino]-1-oxapropyl)-1,4-di-bromobenzene (3). A 100-mL round-bottom flask with a magnetic stir bar was charged with anhydrous potassium carbonate (15.47 g, 111.98 mmol), 2-chlorotriethylamine hydrochloride (4.85 g, 27.99 mmol) and 80 mL acetone. The stirred mixture was purged with argon for 15 min followed by the addition of 2,5-dibromohydroquinone (**1**) (3 g, 11.19 mmol). After 15 min of additional purging, the reaction mixture was refluxed for 2 days at 60 °C. 100 mL of water was then added to dissolve the salts. The product mixture was extracted with dichloromethane (60 mL); the organic phase was washed with 10% aqueous NaOH solution (100 mL) and brine (100 mL); and, dried over magnesium sulfate. The solvent was removed by vacuum evaporation. The crude solid product was recrystallized from methanol/water to obtain white microcrystalline product **3** (2.88 g, 55.13%). ^{1}H NMR (400 MHz, $CDCl_3$): δ = 7.14 (s, 2H), 4.05 (d, 4H), 2.92 (d, 4H), 2.67 (t, 8H), 1.11 (m, 12H) ppm. ^{13}C NMR (75.4 MHz, $CDCl_3$): δ = 150.16, 118.66, 111.04, 69.64, 51.22, 47.81, 12.18.

HRMS (TOF MS EI+) m/z obsd for $C_{18}H_{30}Br_2N_2O_2$: 466.0650 (100%), 464.0670 (46.64%), 465.0652 (20.42%). Purity: 98.9 %.

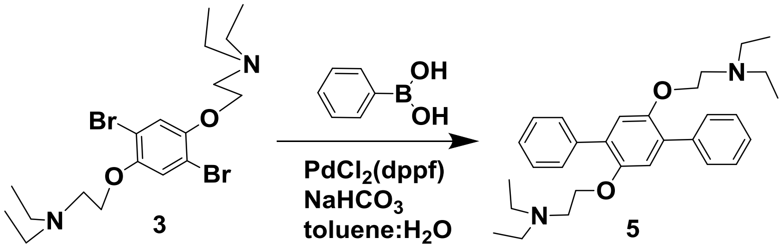

2,5-Bis[3-(N,N-diethylamino)-1-oxopropyl]-1,4-phenylene]-ter-1,4-phenylene (5). **2** (4.00 g, 8.57 mmol), phenylboronic acid (2.08 g, 17.14 mmol), Pd(dppf)Cl_2 (1 mol%) and sodium bicarbonate ($NaHCO_3$) (2.39 g, 28.47 mmol) were added in a 250-mL round bottom flask. A mixture of water (30 mL) and toluene (60 mL) was added to the flask. The mixture was degassed for 4 h, refluxed at 100 °C for 4 days. The end product was washed with water and extracted from dichloromethane. The collected

organic layer was passed through a neutral alumina column to remove the catalyst, followed by drying with $MgSO_4$ and solvent removal under vacuum to afford brown solid **5** (3.08 g, 74.5%). ^{1}H NMR (400 MHz, $CDCl_3$): δ = 7.69 (d, 4H), 7.51 (t, 4H), 7.38 (d, 2H), 7.00 (s, 2H), 4.15 (br, 4H), 2.98 (br, 4H), 2.61 (br, 8H), 1.11 (br, 12H). ^{13}C NMR (150.9 MHz, DMSO-D6: D_2O (2:1)): δ = 149.15, 137.44, 130.25, 129.01, 127.78, 127.16, 115.66, 63.88, 49.70, 47.23, 8.40. HRMS (TOF MS EI+) m/z obsd for $C_{30}H_{40}N_2O_2$: 460.3092 (100%), 461.3142 (16.81%). Purity: 98.6 %.

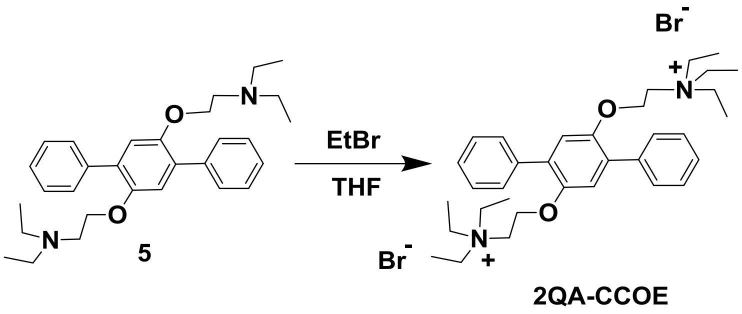

2QA-CCOE. 0.5 g (1.08 mmol) of **5** was dissolved in THF (15 mL). 5 mL of bromoethane was added to the solution under argon environment. The mixture was refluxed at 50 °C for 6 days under continuous stirring. The crude product was filtered and then acetone was added to the filtrate to precipitate the pure product which was collected after centrifugation from acetone, dried under vacuum at 60 °C to afford **2QA-CCOE** (0.53 g, 72.31%) as light yellow product. ^{1}H NMR (400 MHz, DMSO-D6: D_2O (2:1)): δ = 7.58-7.31 (br, 10H), 7.11-6.95 (m, 2 H), 4.16 (br, 14H), 3.44 (br, 2H), 3.07 (br, 6H), 2.82 (br, 4H), 0.97 (br, 12H). ^{13}C NMR (75.4 MHz, DMSO-D6: D_2O (2:1)): δ = 149.99, 137.99, 130.99, 129.88, 128.73, 127.49, 116.31, 63.00, 55.80, 51.08, 10.33. Elemental Analysis calcd: $C_{34}H_{50}Br_2N_2O_2$(%): C 60.18, H 7.43, N 4.13, O 4.72; found: C 60.14, H 6.98, N 4.28, O 4.71.

Detailed synthesis of 4QA-CCOE.

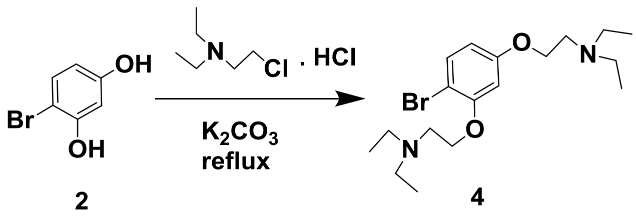

2,4-Bis(3-[N, N-diethylamino]-1-oxapropyl)-bromobenzene (4). A 100-mL round-bottom flask with a magnetic stir bar was charged with anhydrous potassium carbonate (10.97 g, 79.36 mmol), 2-chlorotriethylamine hydrochloride (4.12 g, 23.80 mmol) and 80 mL acetone. The stirred mixture was purged with N_2 for 15 min followed by the addition of 4-bromoresorcinol (**2**) (3 g, 15.87 mmol). After 15

min of additional purging, the reaction mixture was refluxed for 2 days at 60 °C. 100 mL of water was then added to dissolve the salts. The product mixture was extracted with dichloromethane (60 mL); the organic phase was washed with 10% aqueous NaOH solution (2 × 50 mL) and brine (2 × 50 mL); dried over magnesium sulfate and filtered. The solvent was removed by vacuum evaporation to yield crude oily solid which was further recrystallized from methanol/water (2: 1) first and then from ethanol/water (2:1) to yield white microcrystalline product **4** (3.19 g, 52 %). ^{1}H NMR (400 MHz, $CDCl_3$): δ = 7.27 (s, 1H), 7.14 (s, 2H), 4.05 (t, 4H), 2.92 (t, 4H), 2.67 (q, 8H), 1.09 (t, 12H). ^{13}C NMR (75.4 MHz, $CDCl_3$): δ = 158.67, 150.03, 132.41, 118.53, 110.91, 102.47, 101.34, 69.10, 51.39, 47.89, 11.85. HRMS (TOF MS EI+) m/z obsd for $C_{18}H_{31}BrN_2O_2$: 386.1573 (100%), 385.1543 (16.99%), 387.1566 (29.67%), 388.1561 (91.03%). Purity: 98.8 %.

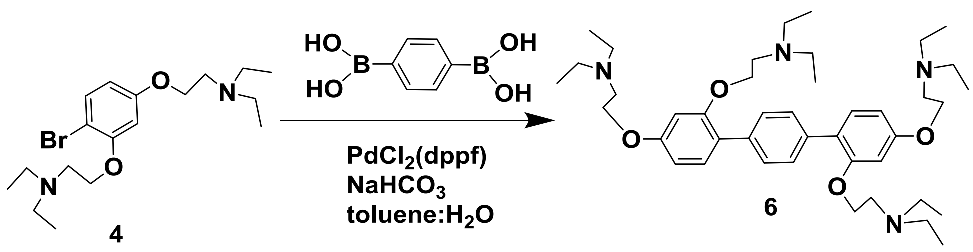

1,3,6,8-tetra[3-N, N-diethylamino]-1-oxapropyl-p-triphenyl (6). **4** (4.69 g, 12.11 mmol), benzene-1,4-diboronic acid (1.00 g, 6.05 mmol), $PdCl_2$(dppf) (1 mol%) and sodium bicarbonate ($NaHCO_3$) (2.39 g, 28.47 mmol) were added in a 25-mL round-bottom flask. A mixture of water (10 mL) and toluene (20 mL) was added to the flask. The mixture was degassed for 4 h, refluxed at 110 °C for 4 days. The end product was washed with water and extracted from dichloromethane. The collected organic layer was passed through a neutral alumina column to remove the extra catalyst, followed by drying with $MgSO_4$ and solvent removal under vacuum to afford **6** as brown solid (3.05 g, 36.44 %). ^{1}H NMR (400 MHz, $CDCl_3$): δ = 7.82 (d, 1H), 7.75 (m, 1H), 7.55 (d, 1H), 7.47 (t, 1H), 7.24 (m, 0.5H), 7.17 (t, 1H), 6.60-6.49 (m, 4.5H), 4.18-3.99 (m, 7H), 2.99-2.85 (m, 5H), 2.70-2.55 (m, 14H), 1.37 (m, 6H), 1.15-0.98 (m, 24H). ^{13}C NMR (150.9 MHz, DMSO-D6: D_2O (2:1)): δ = 158.96, 155.88, 135.54, 130.61, 129.17, 128.14, 105.95, 99.79, 66.29, 68.88, 50.88, 24.38, 11.02. HRMS (TOF MS EI+) m/z obsd for $C_{42}H_{66}N_4O_4$: 691.5161 (100 %). Purity: 98.1 %.

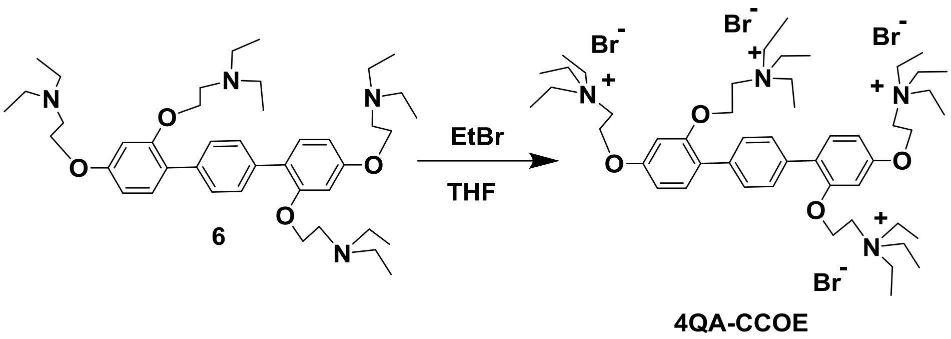

4QA-CCOE. 0.5 g (0.72 mmol) of **6** was dissolved in THF (10 mL). 5 mL of bromoethane was added to the solution under argon environment. The mixture was refluxed for 6 days at 50 °C under continuous stirring. The crude product was filtered and then acetone was added to the filtrate to precipitate the product which was collected after centrifugation from acetone, dried under vacuum at 60 °C to afford **4QA-CCOE** (0.55 g, 95%) as light yellow product. ^{1}H NMR (400 MHz, DMSO-D6: D_2O (2:1)): δ = 7.76 (d, 1H), 7.78-7.71 (br, 0.5H), 7.55 (m, 1H), 7.44 (d, 1H), 7.35-7.28 (m, 2H), 6.87 (d, 0.5H), 6.83-6.74 (m, 1H), 6.72-6.63 (m, 3H), 4.50 (br, 7.5H), 3.70 (m, 7.5H), 3.41 (br, 19H), 3.11 (m, 4H), 1.56 (br, 29H), 1.21 (br, 9H). ^{13}C NMR (150.9 MHz, DMSO-D6: D_2O (2:1)): δ = 159.29, 156.00, 134.63, 131.34, 129.90, 128.88, 108.53, 102.17, 61.89, 55.93, 53.88, 25.11, 7.56. Elemental Analysis calcd: $C_{50}H_{85}$ $Br_4N_4O_4$ (%): C 53.34, H 7.61, N 4.98, O 5.68; found: C 53.61, H 7.82, N 4.70, O 5.46.

Detailed synthesis of CCPE.

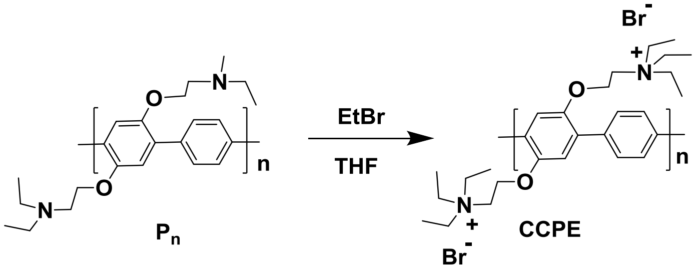

CCPE. Poly{[2,5-bis(2-(N,N-diethylamino)ethoxy)-1,4-phenylene]-alt-1,4-phenylene} (**P_n**) (0.3 g, 0.756 mmol RU) was quaternized using bromoethane following the procedure in the literature.[5] Briefly, **P_n** was dissolved in THF at 50 °C under argon environment. After 30 min, bormoethane (3 mL) and dimethylsulfoxide (DMSO) (3 mL) were added to the flask and stirred at 50 °C for 5 days. The polymer was precipitated using acetone. The precipitate was separated by centrifugation and dried under vacuum at 50 °C overnight, yielding CCPE (0.21 g, 43.65%) as a brown solid.^{1}H NMR (400 MHz, DMSO-D6: D_2O (2:1)): δ = 7.72 (br, 4H), 7.12 (br, 2H), 3.30 (br, 4H), 3.18 (br, 4H), 2.97 (br, 4H), 2.53 (br, 12H),

1.02 (br, 14H). Elemental Analysis calcd: $C_{28}H_{46}Br_2N_2O_2$ (%): C 55.82, H 7.70, N 4.65, O 5.31; found: C 55.60, H 7.52, N 4.78, O 5.49.

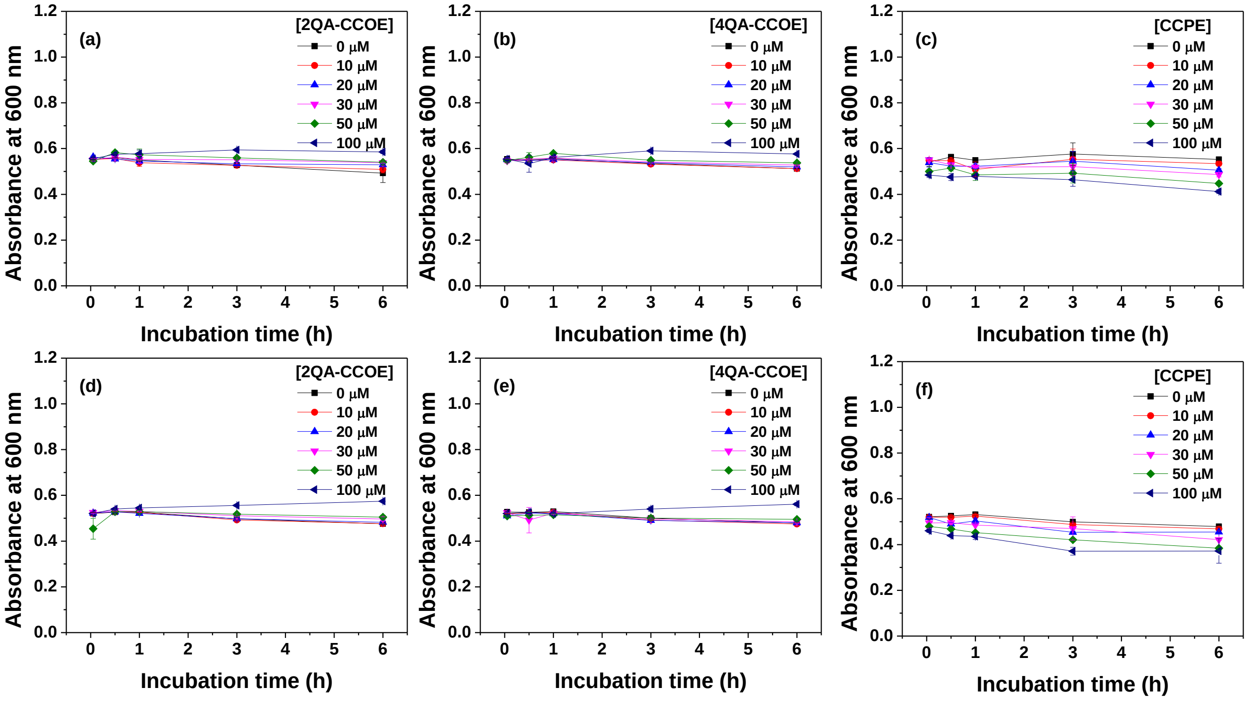

Figure S1: UV/Vis absorbance of wild-type (top panel; a-c) and amp-resistant (bottom panel; d-f) *E. coli* suspension (in 5 mM PBS) at 600 nm upon treatment with 2QA-CCOE (a, d) and 4QA-CCOE (b, e) and CCPE (c, f). Each data point represents the mean and standard deviation of 3 replicates.

Two-step-treat (LB), then grow (LB) process (treat (LB), then grow (LB)) (Supplementary Fig. S2), and, one-step treat and grow in LB media (treat and grow (LB)) (Supplementary Fig. S3). In the one-step "treat and grow (LB)" process, where *E. coli* cells were treated and grown simultaneously in LB media for 0-6 h, we saw a significant increase in absorbance of *E.coli* over time (Supplementary Fig. S3) and minimal % growth inhibition (Supplementary Table S2) suggesting bacterial growth during treatment with conjugated molecules as opposed to "treat (PBS), then grow (LB)" process (Fig. 4). In the one-step process, cell division and coating/intercalation of conjugated molecules may compete with each other. Since the % growth inhibition was low, we attempted to quantify whether the bacterial suspension had enough CCPE chains to effectively coat the bacteria multiplied over time. We incubated bacteria cells in different concentrations of CCPE. After incubation for a certain time, we centrifuged the suspension and measured the fluorescence of the supernatant (containing unbound CCPE). At CCPE concentration ≥ 50

μM, the maximum adsorption of CCPE on the bacterial surface was achieved (~ 98%, Supplementary Fig. S4), based on very low fluorescence of the supernatant. Based on the known cell counts of *E. coli* in this suspension and the CCPE concentration corresponding to maximum bacterial surface coverage, we estimated that ~6.47 x10^5 chains of CCPE, on an average, were adsorbed by a single bacterial cell. As per Supplementary Fig. S3, the *E. coli* cells doubled within 1 h of treatment in the one-step process. Based on these observations, these cells doubled would require 2.07×10^{14} chains of CCPE/mL. By using 100 μM CCPE in the treatment system, we offered 1.06×10^{15} chains of CCPE/mL which is ~5 times higher than what would have been needed for achieving the most efficient coating of the cells. Despite offering excess conjugated molecules, we saw rapid cell division as opposed to "treat (PBS), then grow (LB)" process.

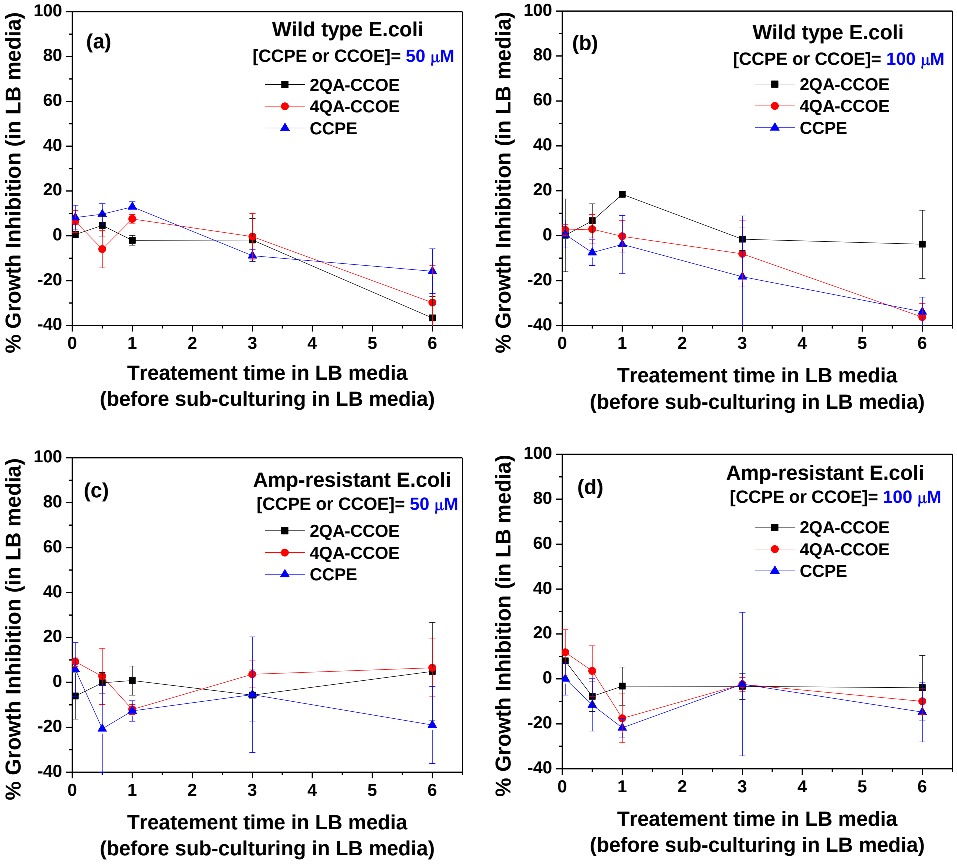

Figure S2: % growth inhibition of wild-type (a, b) and amp-resistant (c, d) *E. coli* grown in LB media for "treat (LB), then grow (LB)" process. In this process, *E. coli* cells were treated first for 0-6 h in 50 μM (a, c) and 100 μM (b, d) of 2QA-CCOE, 4QA-CCOE, or CCPE in LB media. The cells were then transferred and allowed to grow in a fresh LB media for 3 h. Each data point represents the mean and standard deviation of 3 replicates.

This suggests that not having enough CCPE (to coat bacteria) is not the root cause of poor growth inhibition. We thus hypothesized that the poor % growth inhibition in "treat (LB), then grow (LB)" and the one-step method was due to high ionic strength of LB media (171 mM) used which led to charge screening of both bacteria and cationic conjugated molecules and minimized their electrostatic interactions. To test this hypothesis, we incubated *E. coli* in 5 mM PBS buffer for 1 h without CCPE, then centrifuged and resuspended the cells in water. This yielded the zeta potential of bacteria as -40 mV (Fig. 6). This value was similar to what was reported for *E.coli* incubated in electrolyte solution with similar ionic strength (8.4 mM).[6] On the other hand, the zeta potential of an electrolyte solution with ionic strength similar to our LB media (168 mM) was reported as -20 mV.[6] These results suggested the importance of ionic strength of media on the antimicrobial and/or grown inhibition efficacy. Our future work will focus on more systematic studies along this direction. The zeta potential of the bacteria changed when those were treated with different concentrations of conjugated molecules.

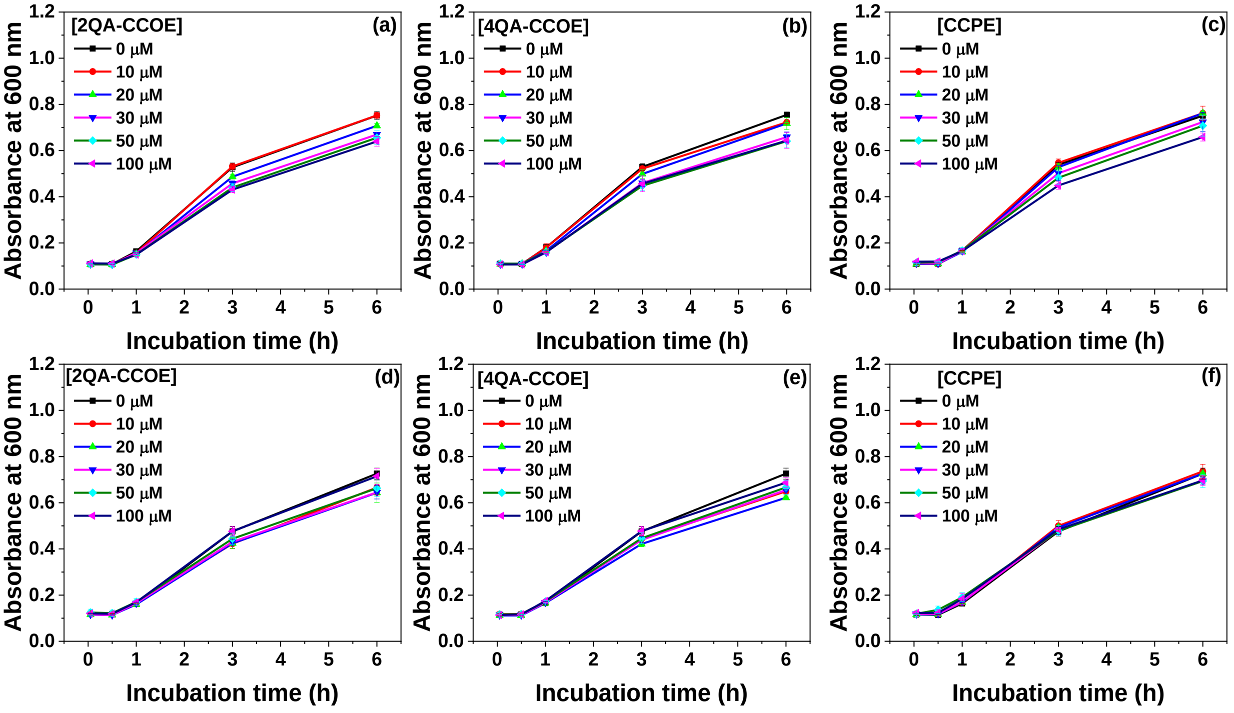

Figure S3: UV/Vis absorbance (at 600 nm) of wild-type (top panel; a-c) and amp-resistant (bottom panel; d-f) *E. coli* suspension in LB media (ionic strength = 171 mM) in the presence of 2QA-CCOE (a, c), 4QA-CCOE (b, e), and CCPE (c, f) in the LB media ("treat and grow (LB)" process). While *E. coli* cells were being incubated in CCOE/CCPE in LB media in this "treat and grow (LB)" process, the absorbance of *E. coli* was measured at certain interval for 6 h. Each data point represents the mean and standard deviation of 3 replicates.

Table S2. % Growth inhibition of wild-type and amp-resistant *E. coli* in LB media in the presence of 2QA-CCOE, 4QA-CCOE and CCPE in the LB media (ionic strength = 171 mM) ("treat and grow (LB)" process). The concentrations of CCOE/CCPE were 50 μM and 100 μM. While the *E.coli* cells were being incubated in CCOE/CCPE in LB media over 6 h in this "treat and grow (LB)" process, the corresponding % growth inhibition values were calculated after 3 h and 6 h.

CCPE/CCOE	*Incubation and growth time (h)*	*[CCPE or CCOE] (μM) in LB media*	*% Growth Inhibition ± standard deviation*	
			Wild type E. coli	*Amp resistant E. coli*
2QA-CCOE	*3*	*50*	*20.9 ± 4.0*	*11.2 ± 6.8*
		100	*24.0 ± 3.2*	*1.0 ± 6.0*
	6	*50*	*14.4 ± 4.7*	*11.7 ± 5.9*
		100	*17.5 ± 3.2*	*2.6 ± 5.7*
4QA-CCOE	*3*	*50*	*19.6 ± 4.9*	*8.4 ± 6.5*
		100	*16.7 ± 8.1*	*-1.0 ± 5.6*
	6	*50*	*18.3 ± 4.5*	*9.8 ± 5.1*
		100	*17.4 ± 5.2*	*5.9 ± 3.6*
CCPE	*3*	*50*	*14.4 ± 6.7*	*0 ± 6.1*
		100	*23.2 ± 3.39*	*-0.7 ± 2.6*
	6	*50*	*7.3 ± 7.1*	*9.7 ± 7.9*
		100	*15.6 ± 2.7*	*9.9 ± 6.1*

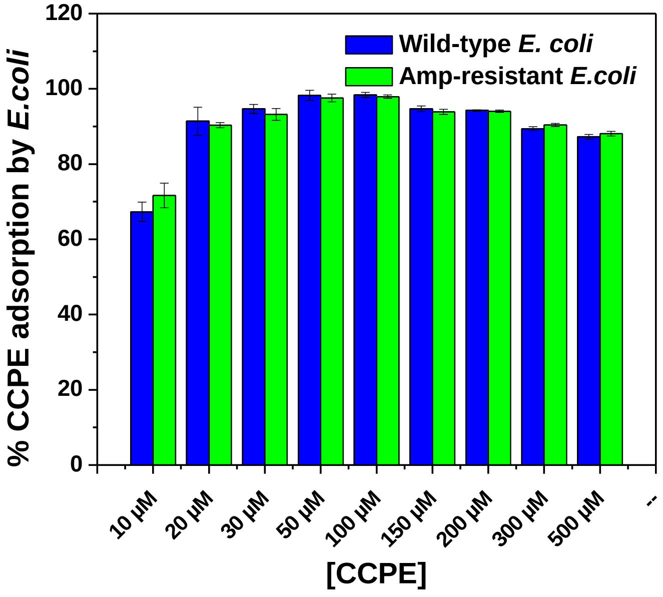

Figure S4: % CCPE adsorption by *E. coli* as a function of concentration of CCPE after incubation for 1 h in 5 mM PBS.

CFU of CCOE/CCPE treated wild-type and amp-resistant E.coli.

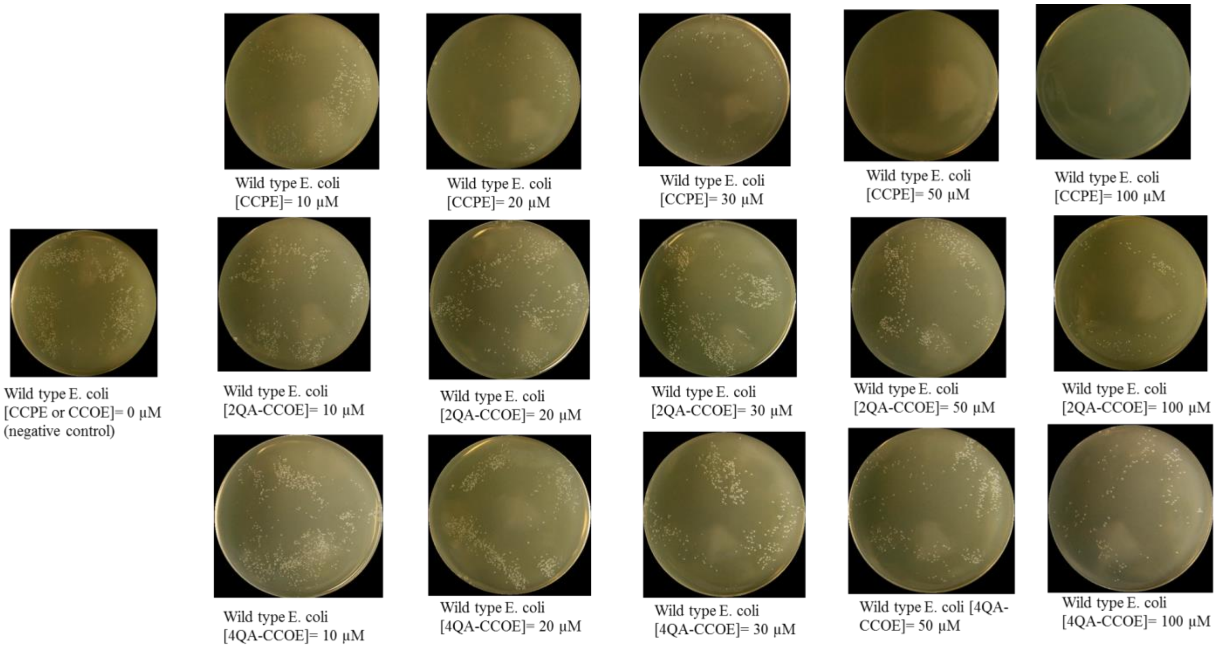

Figure S5: CFU reduction of wild-type *E. coli* grown on agar plates after treatment with CCOEs/CCPE.

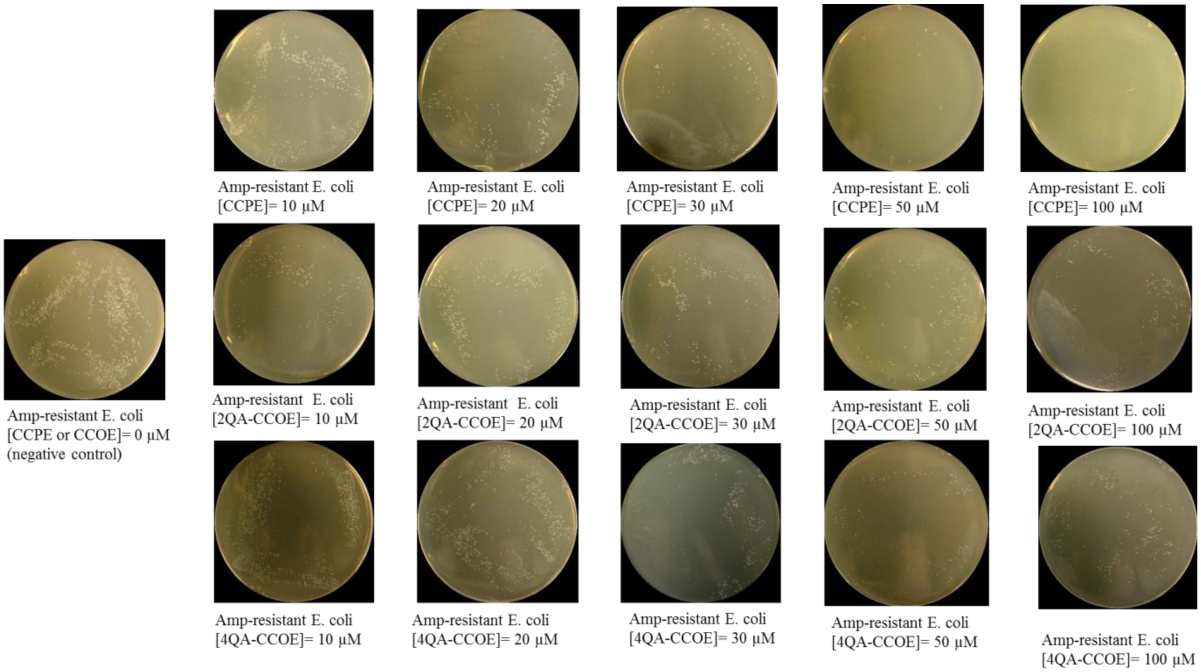

Figure S6: CFU reduction of amp-resistant *E. coli* grown on agar plates after treatment with CCOEs/CCPE. Agar plates were supplemented with ampicillin.

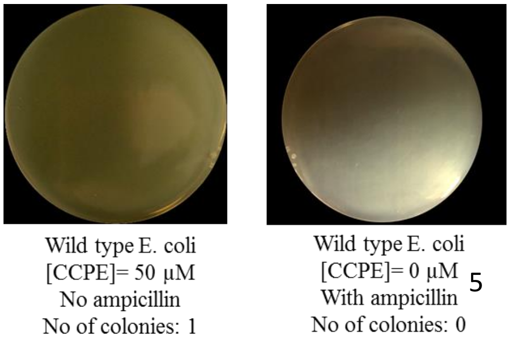

Figure S7: CFU reduction of pretreated (with 50 µM CCPE) wild-type E.coli grown on ampicillin-free agar plate (left); untreated, wild-type *E. coli* grown on agar plate containing 100 µg/mL ampicillin (right).

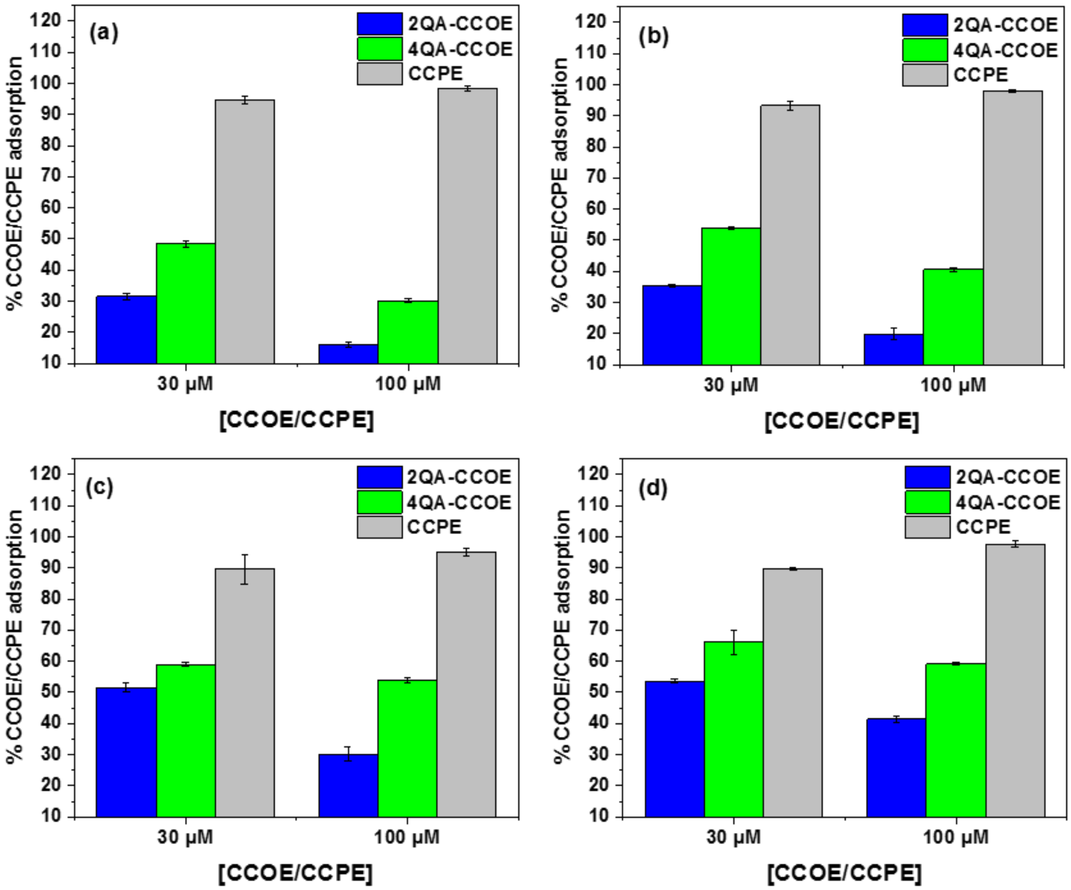

Figure S8: CCOE and CCPE mass adsorption (%) on wild-type (a, c) and amp-resistant (b, d) *E. coli* suspension after treatment with CCOEs/CCPE for 1 h (a, b) and 6 h (c, d). Each data point represents the mean and standard deviation of 3 replicates.

CCOE/CCPE adsorption. The percent (%) mass adsorption of conjugated molecules on the outer cell envelope of *E. coli* during 1 h (Supplementary Fig. S8a, b) and 6 h (Supplementary Fig. S8c, d) treatment was calculated using equation (3). Higher % CFU reduction by CCPE treatment (Fig. 5) was rationalized in prior discussion with a higher possibility of CCPE adsorption and Supplementary Fig. S8 provides evidence to support that. During 1-h treatment with 30 μM solution (Supplementary Fig. S8), ≥ 95% of the CCPE in the treatment solution was adsorbed by wild-type *E.coli*, while 4QA-CCOE (48%, Supplementary Fig. S8a) and 2QA-CCOE (31%, Supplementary Fig. S8a) were adsorbed less. A similar extent of CCOE adsorption was reported by Bazan.[7] While the low adsorption of 2QA-CCOE was attributed to low water solubility and poor electrostatic interaction with charged *E. coli* surface, the reason behind the not-so-high adsorption of 4QA-CCOE was likely a result of a competition between high water solubility and hydrophilic-hydrophobic interactions with bacteria.[7] If the chain length of an electrolyte is small, but highly water-soluble, the electrolyte may tend to remain in the solution rather than attaching to the bacterial surface. These observations point towards the importance of the right balance between hydrophilicity and hydrophobicity of conjugated molecules to make the interactions with biomolecules efficient.

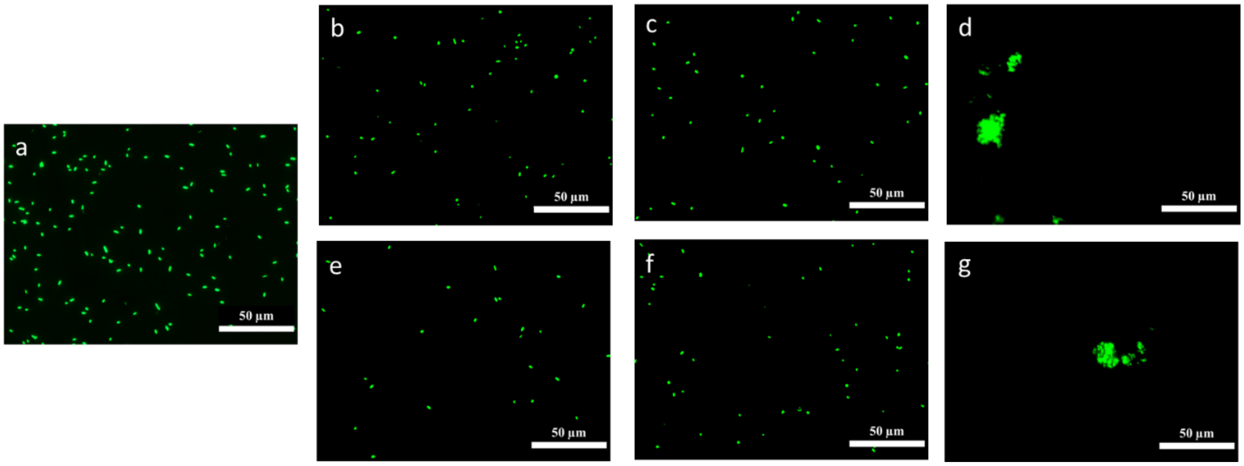

Figure S9: Fluorescence microscopy images of GFP-expressing amp-resistant *E. coli* without CCPE/CCOE treatment (a); and treated with 30 μM (b-d) and 100 μM (e-g) 2QA-CCOE (b, e), 4QA-CCOE (c, f), and CCPE (d, g) for 1 h. The scale bar is 50 μm. CCPE treated cells aggregated, while CCOEs did not cause aggregation.

Bacterial Culture (Selection process to get amp-resistant E.coli).

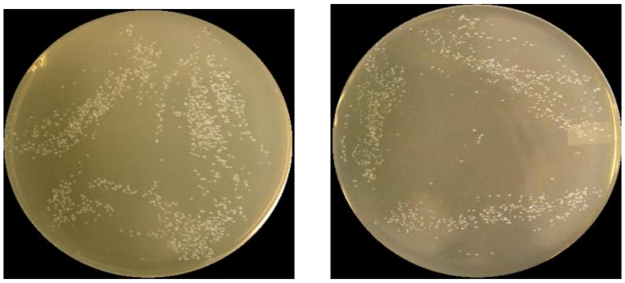

Figure S10: CFUs of untreated amp-resistant *E. coli* grown on agar plates made of LB supplemented with (left) and without (right) ampicillin. The colony counts appeared to be similar.

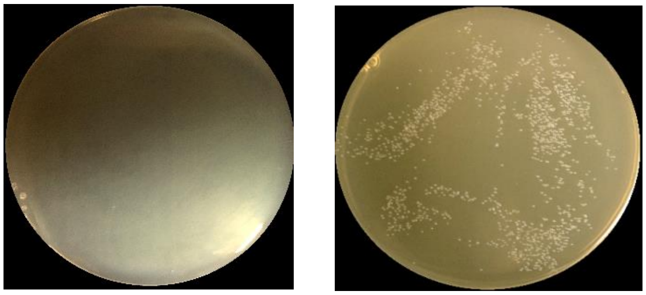

Figure S11: CFUs of untreated wild-type (left) and amp-resistant (right) *E. coli* grown on agar plates made of LB supplemented with ampicillin. Wild-type bacteria did not grow on LB media supplemented with ampicillin, while amp-resistant E. coli cells grew on the media.

References.

1. Lee, T. S. *et al.* BglBrick vectors and datasheets: A synthetic biology platform for gene expression. *J. Biol. Eng.* **5,** 1–14 (2011).

2. Tu, Q. *et al.* Room temperature electrocompetent bacterial cells improve DNA transformation and recombineering efficiency. *Sci. Rep.* **6,** 24648-1–8 (2016).

3. Fu, C., Donovan, W. P., Shikapwashya-Hasser, O., Ye, X. & Cole, R. H. Hot fusion: An efficient method to clone multiple DNA fragments as well as inverted repeats without ligase. *PLoS One* **9,** 1–20 (2014).

4. Kovach, M. E. *et al.* Four new derivatives of the broad-host-range cloning vector pBBR1MCS, carrying different antibiotic-resistance cassettes. *Gene* **166,** 175–176 (1995).

5. Balanda, P. B., Ramey, M. B. & Reynolds, J. R. Water-soluble and blue luminescent cationic polyelectrolytes based on poly(p-phenylene). *Macromolecules* **32,** 3970–3978 (1999).

6. Walczak, J. J. *et al.* The Effects of Starvation on the Transport of Escherichia coli in Saturated Porous Media are Dependent on pH and Ionic strength. *Colloids Surf B Biointerfaces* **90,** 129–136 (2012).

7. Yan, H. *et al.* Influence of molecular structure on the antimicrobial function of phenylenevinylene conjugated oligoelectrolytes. *Chem. Sci.* **7,** 5714–5722 (2016).
